# Novel-Substituted Heterocyclic GABA Analogues. Enzymatic Activity against the GABA-AT Enzyme from *Pseudomonas fluorescens* and In Silico Molecular Modeling

**DOI:** 10.3390/molecules23051128

**Published:** 2018-05-09

**Authors:** Erika Tovar-Gudiño, Juan Alberto Guevara-Salazar, José Raúl Bahena-Herrera, José Guadalupe Trujillo-Ferrara, Zuleyma Martínez-Campos, Rodrigo Said Razo-Hernández, Ángel Santiago, Nina Pastor, Mario Fernández-Zertuche

**Affiliations:** 1 Instituto de Investigación en Ciencias Básicas y Aplicadas, Centro de Investigaciones Químicas, Universidad Autónoma del Estado de Morelos, Cuernavaca 62209, Morelos, Mexico; egt@uaem.mx (E.T.-G.); zmc@uaem.mx (Z.M.-C.); 2 Departmento de Bioquímica, Escuela Superior de Medicina, Instituto Politécnico Nacional, Cd Mexico 11340, Mexico; jguevaras@ipn.mx (J.A.G.-S.); joseraul.bahe@gmail.com (J.R.B.-H.); jtrujillo@ipn.mx (J.G.T.-F.); 3 Instituto de Investigación en Ciencias Básicas y Aplicadas, Centro de Investigación en Dinámica Celular, Universidad Autónoma del Estado de Morelos, Cuernavaca 62209, Morelos, Mexico; rodrigo.razo@uaem.mx (R.S.R.-H.); jast@uaem.mx (Á.S.); nina@uaem.mx (N.P.)

**Keywords:** heterocyclic GABA analogues, GABA-AT inhibitors, GABA-AT docking, molecular electrostatic potential

## Abstract

γ-Aminobutyric acid (GABA) is the most important inhibitory neurotransmitter in the central nervous system, and a deficiency of GABA is associated with serious neurological disorders. Due to its low lipophilicity, there has been an intensive search for new molecules with increased lipophilicity to cross the blood-brain barrier to raise GABA concentrations. We have designed and evaluated in vitro and in silico some new analogues of GABA, where the nitrogen atom at the γ-position is embedded in heterocyclic scaffolds and determined their inhibitory potential over the GABA-AT enzyme from *Pseudomonas fluorescens*. These modifications lead to compounds with inhibitory activity as it occurs with compounds **18a** and **19a**. The construction of *Pseudomonas fluorescens* and human GABA-AT models were carried out by homology modeling. Docking assays were done for these compounds over the GABA-AT enzyme models where **19a** showed a strong interaction with both GABA-AT enzymes.

## 1. Introduction

**Figure 1:**
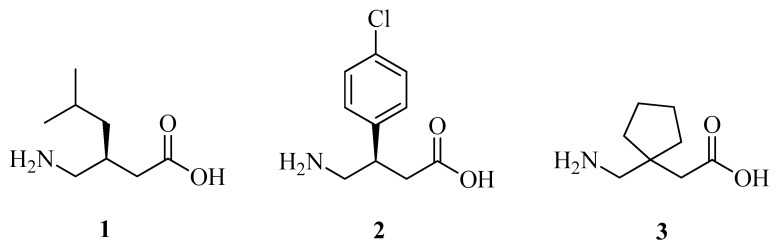
GABA analogues with clinical applications.

γ-Aminobutyric acid (GABA) is the most important and most abundant inhibitory neurotransmitter in the central nervous system. This inhibitory activity is expressed through an interaction with the receptor subtypes GABA_A_, GABA_B_ and GABA_C_ [1]. GABA concentration is regulated by two enzymes, l-glutamate decarboxylase (GAD) [2] and the catabolic enzyme GABA aminotransferase (GABA-AT). The former catalyzes the decarboxylation of l-glutamate, resulting in the biosynthesis of GABA. The latter degrades GABA to succinic semi-aldehyde throughout a reversible transamination reaction [3,4]. A decrease in GABA concentration in the brain has been associated with some common neurological disorders such as epilepsy [5], Alzheimer’s [6], Parkinson’s [7], Huntington’s [8] as well as tardive dyskinesia diseases [9]. However, direct administration of GABA is not an efficient therapy; due to its very low lipophilic character, this compound cannot cross the blood-brain barrier [10]. Therefore, it is not surprising that an intensive search for new analogues with increased lipophilicity has been carried out by several research groups. Figure 1 shows some representative GABA analogues used in the clinic for a range of CNS disorders, such as (*S*)-pregabalin (**1**), (*R*)-baclofen (**2**) and gabapentin (**3**).

(*S*)-Pregabalin (**1**) is an analog of GABA that binds to the α2-δ protein [11,12], a voltage-dependent calcium channel, reducing the release of excitatory neurotransmitter and peptide modulators which may be responsible for its anticonvulsant, anxiolytic and analgesic effects [13,14,15]. (*R*)-Baclofen (**2**) is a GABA_B_ agonist used clinically to treat spasticity and to improve mobility of patients with multiple sclerosis and other injuries of the spinal cords, relieving pain and muscle stiffness [16,17]. Gabapentin (**3**) is a neuroleptic drug used clinically for the prevention of seizures, anxiety, tardive dyskinesia and neuropathic pain [18,19,20]. The synthesis of analogues of **1** and **2** based on isosteric replacements or carbon chain homologations have also been reported in the literature [21,22,23,24,25,26,27]. Wanner has reported a series of *N*-alkylated pyrrolidine-2-yl acetic acids as potential GABA transport inhibitors [28] and Krogsgaard-Larsen, the synthesis of (*R*)- and (*S*)-homo-β-proline and piperidine derivatives as GABA_A_ and GABA_B_ agonists [29]. Some other GABA derivatives with improved lipophilic character include amides of GABA with fatty acids [30], linoleic, stearic and palmitic derivatives [31], amides of nicotinic acid [32], Schiff bases of GABA [33] esters with cholesterol [34], *N*-acyl-GABA derivatives [35] and enolic 1,3-cyclohexandione analogues of GABA [36].

**Figure 2:**
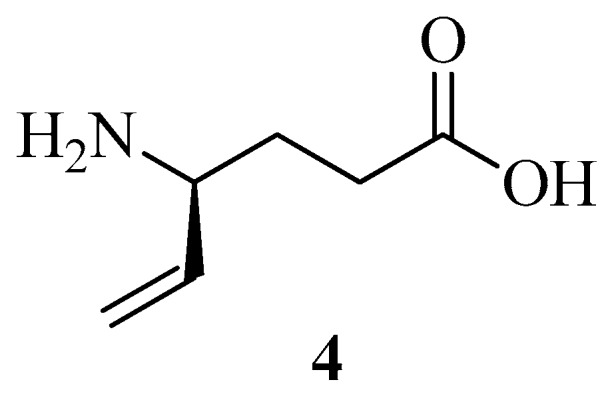
Vigabatrin, an inactivator of GABA-AT.

One strategy used to raise GABA concentration in the brain has been the design of GABA analogues to inactivate GABA-AT [3]. In this regard, vigabatrin (**4**, Figure 2) is one of the first reported GABA-AT inhibitors [37].

Silverman and co-workers have designed, synthesized and evaluated a variety of GABA-AT inhibitors. These inhibitors include heteroaromatic substrates [38,39], fluorinated and conformationally restricted fluorinated analogues [40,41,42], bioisosters of carboxylic acids [43], and some tetrahydrothiophene analogues and their inactivation mechanism [44,45].

**Figure 3:**
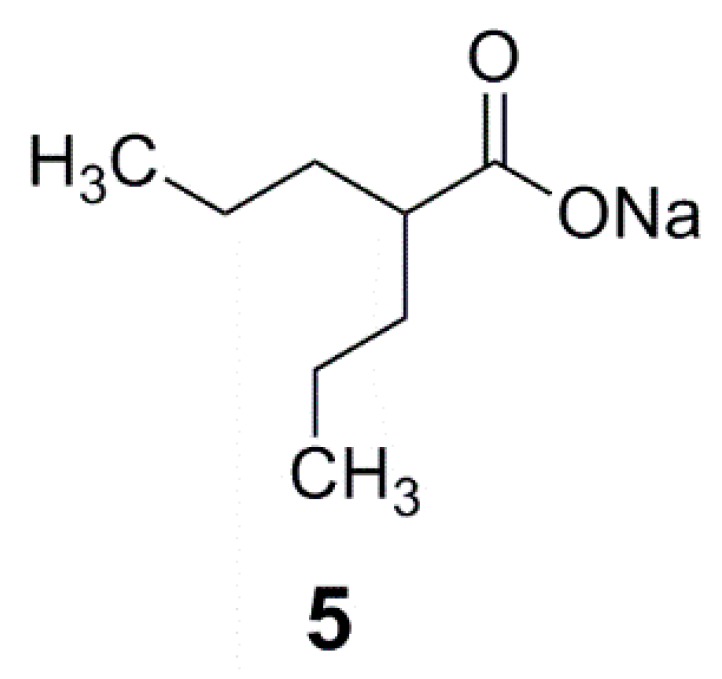
Sodium valproate VPNa **5**, an antiepileptic agent.

Bansal has reported a series of hydrazone [46] and Schiff bases [47] as GABA analogues; Conti has also reported some bicyclic-γ-amino acids as potent GABA-AT inhibitors [48]. Among other type of compounds not related to the GABA framework, sodium valproate (**5**, Figure 3) is a well-known antiepileptic compound [49]. Although its mode of action has not been well-established, its antiepileptic properties have been attributed, at least in part, to an inhibition of the GABA-AT enzyme [50].

We present in this study the synthesis of new GABA analogues where the nitrogen atom at the γ-position is embedded in heterocyclic scaffolds such as **9a**–**c** and **18**–**21** as well as their in vitro and in silico activity as GABA-AT inhibitors. These analogues maintain the GABA backbone and possess a β-alkyl or β-aryl substituent in analogy to (*S*)-pregabalin **1** or (*R*)-baclofen **2** in order to improve the lipophilic character of these compounds. Contrary to what happens in the case of vigabatrin **4**, and other GABA analogues with primary amine groups that allow them to react with the aldehyde group of the PLP cofactor of the GABA-AT enzyme [3], the inclusion of the nitrogen atom in these non-aromatic heterocyclic systems makes them tertiary amines with higher basicity (pK_b_~4.2). This increased basicity favors the transfer of the acidic carboxylic proton to the nitrogen atoms generating zwitterionic species that may facilitate their interaction with the GABA-AT enzyme. We show in this work, that these structural modifications may lead to compounds with GABA-AT inhibitory activity as it occurs with compounds **18a** and **19a**. We report the inhibitory potential of these compounds toward the GABA-AT enzyme, as well as computational studies regarding molecular similarity analysis and molecular docking studies to explain their inhibitory character based in different structural and electronic parameters, and their interaction with the *Pseudomonas fluorescens* GABA-AT. In addition, we also carried out a molecular docking study over a homology modeled human GABA-AT enzyme to identify compounds as potential candidates for future in vivo studies.

## 2. Results and Discussion

### 2.1. Chemistry

**Scheme 1:**
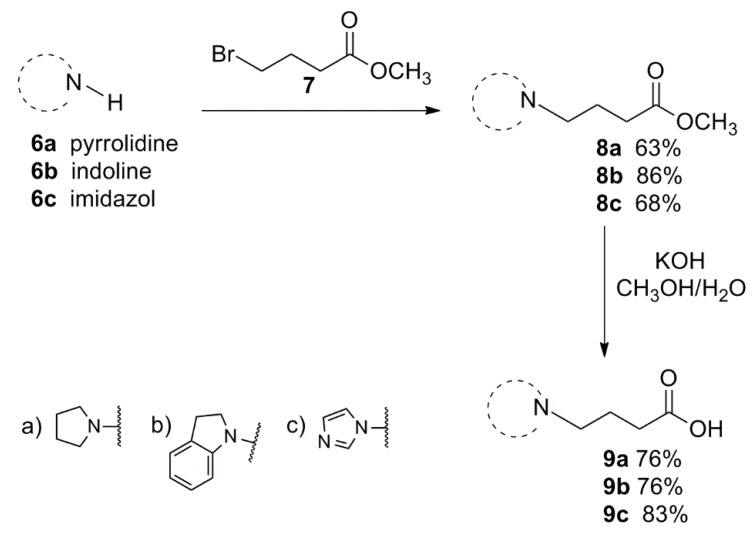
Synthesis of analogues **9a**–**c**.

The synthesis of compounds **9a**–**c** is outlined on Scheme 1. The *N*-alkylation reaction of heterocycles **6a**–**c** with methyl-4-bromobutanoate (**7**) was accomplished in the absence of both solvent and base to afford esters **8a**–**c** in good yields.

For compounds **6a** and **6b**, the reactions were run at room temperature. For **6c** the reduced nucleophilic character of the nitrogen imidazole required temperatures around 70 °C to produce **8c** in a reasonable yield. In a subsequent step, basic hydrolysis with potassium hydroxide of **8a**–**c** afforded the corresponding acids **9a**–**c**.

**Scheme 2:**
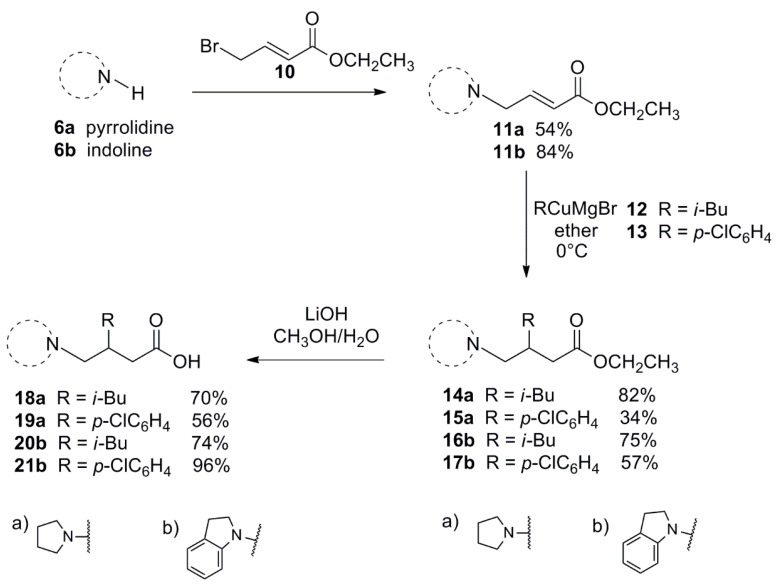
Synthesis of β-substituted analogues **18**–**21**.

Scheme 2 shows the synthesis of analogues **18**–**21**. As in the previous case, the *N*-alkylation reaction of **6a** or **6b** with commercially available methyl-4-bromocrotonate (**10**) was carried out at room temperature to afford the α,β-unsaturated esters **11a** and **11b** with 54% and 84% yields, respectively.

The second step in the synthesis involved the conjugate addition of cuprates **12** and **13** in order to introduce the isobutyl or *p*-Cl-C_6_H_4_ substituents of analogues **18**–**21**. Cuprate **12** was generated from isobutyl magnesium bromide and CuI in ether at −78 °C and added to the conjugated esters **11a** and **11b** at 0 °C to afford **14a** and **16b** in very good yields. On the other hand, cuprate **13** was generated from bromochlorobenzene following the same reaction conditions. The conjugate addition of **13** to the unsaturated esters **11a** and **11b** afforded the corresponding products **15a** and **17b** albeit in lower yields. Finally, alkaline hydrolysis of esters **14**–**17** with lithium hydroxide yielded the β-substituted analogues **18**–**21**.

### 2.2. GABA-AT Inhibition

The enzymatic assay for GABA-AT inhibition is based on the role of this enzyme in the degradation of GABA to succinic semialdehyde, and its subsequent transformation to succinate. This involves the concomitant conversion of β-NADP^+^ to β-NADPH by a succinic-semialdehyde dehydrogenase-coupled reaction [51,52,53]. This is evidenced by the absorbance change at 340 nm, corresponding to the formation of β-NADPH from β-NADP^+^, which is proportional to GABA-AT activity.

**Figure 4:**
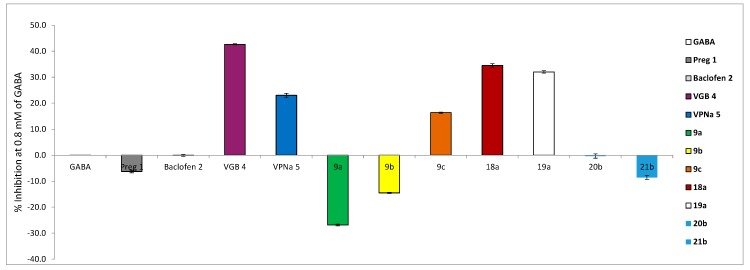
GABA-AT % inhibition of compounds **9a**–**c** and **18**–**21** as compared to VGB **4** and VPNa **5**. * *p* < 0.05: Significant difference comparing GABA with **9a**–**c** and **18**, **19**. * *p* < 0.05: Significant difference comparing VGB **4** with **9a**–**c** and **20**, **21**. * *p* < 0.05: Significant difference comparing VPNa **5** with **9a**–**b** and **18**, **20**, **21**. Comparisons were made by the one-way ANOVA test and Duncan’s means analysis test.

Figure 4 shows the in vitro GABA-AT inhibition assays for compounds **9a**–**c** and **18**–**21** as racemic mixtures, using VGB **4** and VPNa **5** as positive controls. As can be seen in Figure 4 each molecule displays different degrees of inhibition at a constant substrate (GABA) concentration of 0.8 mM.

As can be seen in Figure 4, Pregabalin (**1**) and Baclofen (**2**) show no inhibitory activity against the enzyme. However, **18a** and **19a** showed an inhibitory activity of 35.51 and 32.03%, respectively, with no significant difference with respect to VGB **4** (42.6%). However, with respect to VPNa **5** (23%), compound **18a** shows a significant difference (35%) whereas compound **19a** shows no significant difference (32%) in their inhibitory activity. This inhibitory activity may be associated with the presence of the substituents at C-3, the isobutyl group of **18a** and the *p*-chlorophenyl ring system of **19a** [54]. On the other hand, compounds **9a**, **9b**, **20b** and **21b** showed no significant difference with GABA indicating that they have no activity as inhibitors. The greater size of **20b** and **21b** of these molecules may diminish the degree of affinity at the active site of the enzyme. We have corroborated this assumption based on the molecular docking calculations in the computational studies section.

### 2.3. Computational Studies

#### 2.3.1. Conformational Analysis and Geometric Optimization

The optimized structures of Pregabalin **1**, Baclofen **2**, VGB **4**, VPNa **5** and analogues **9a**, **9b**, **18a**, **19a**, **20b** and **21b** are shown in Figure S19. All these structures correspond to minimum energy structures on the potential surface because their vibrational frequencies were positive.

#### 2.3.2. Natural Partial Charges

Nitrogen and oxygen atoms are susceptible to protonation at physiological pH and their biological activity is directly related to their ability to transfer a proton from the carboxylic function to the nitrogen atom in the heterocyclic ring. To evaluate these effects, the partial charges of the nitrogen and oxygen atoms were evaluated. Table 1 shows the natural partial charges type of the nitrogen and oxygen atoms of all analogues as well as the reference compounds pregabalin **1**, baclofen **2**, VGB **4** and VPNa **5**, in their neutral charge form.

Comparing the nitrogen atom charges (Table 1), the active analogues **19a** and **18a** have slightly more negative partial charge values compared to the non-active analogues **20b** and **21b**. In addition, the nitrogen atoms of VGB **4**, Baclofen **2** and Pregabalin **1** present a more negative partial charge value.

Based on this, we suggest that analogues with a partial charge value more negative than −0.548 in the nitrogen atom will display GABA-AT inhibitory activity, while analogues with less negative partial charge will not show inhibitory activity. On the other hand, **1** and **2** showed more negative partial charges on their nitrogen atoms, but they were inactive inhibiting GABA-AT. Therefore, we propose that for a compound to be active its partial charge value in the nitrogen must be between −0.548 and −0.878. At physiological pH, these nitrogen atoms will possess a hydrogen atom from the carboxylic moiety, increasing their positive partial charge; this makes the protonated nitrogen a better hydrogen bond donor (HBD), which could interact more strongly with a negatively charged residue (Glu or Asp are excellent hydrogen bond acceptors). On other hand, there are no significant differences in the oxygen partial charges among these compounds.

#### 2.3.3. Electrostatic Potential Maps

**Figure 5:**
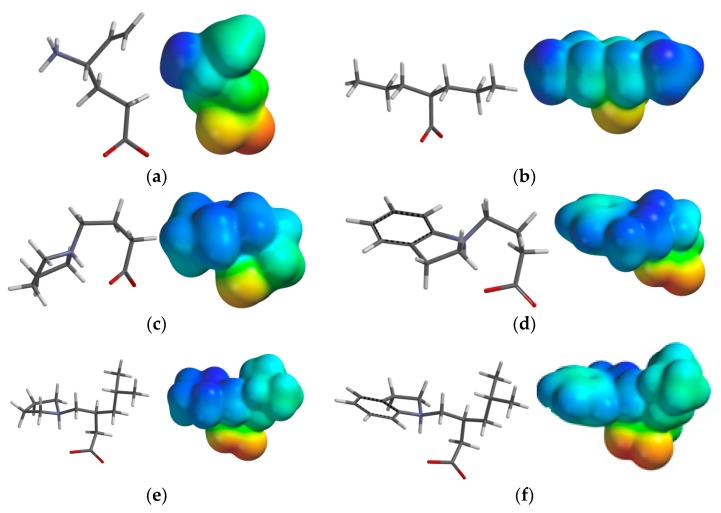

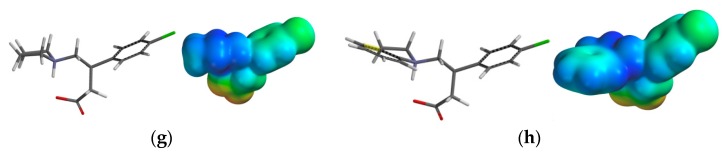
Optimized structures and MEP of all analogues under study in their Zwitterionic form. Red, blue and green color indicates negative, positive and neutral charged regions, respectively. (**a**) (*S*)–VGB **4**; (**b**) VPNa **5** (**c**) **9a**; (**d**) **9b**; (**e**) **18a**; (**f**) **20b**; (**g**) **19a**; (**h**) **21b**. MEP values bars are shown.

Figure 5a–h show the electrostatic potential maps and the optimized structures of all analogues under study in their zwitterionic form. From Figure 5a–h, it can be observed that analogues **18a**, **19a**, **20b** and **21b** have a similar Y-shape structure conformation, whereas analogues **9a**–**c** present a distorted U-shape form. The form of the active (**18a** and **19a**) and the non-active (**20b** and **21b**) analogues is very similar to the conformation of VGB **4** and VPNa **5**. However, when the electrostatic potential maps are compared, it can be observed that analogues **18a** and **19a** have greater similarity in the electronic distribution with VGB **4** and VPNa **5**, whereas the electronic distribution of analogues **20b** and **21b** is different. This is related to the concept of molecular similarity, which states that “molecules with similar structure will have similar activity” [55].

**Figure 6:**
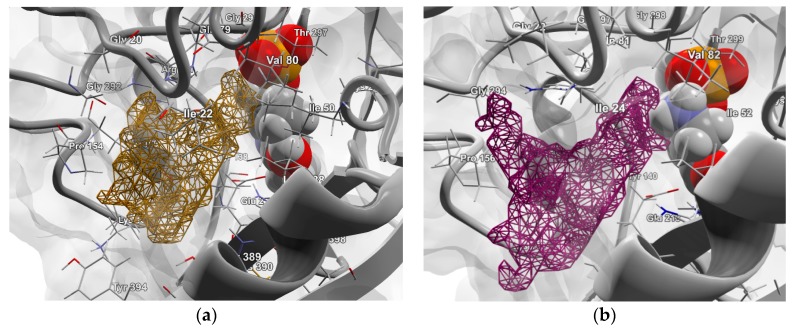
(**a**) GABA-AT catalytic site cavity (in orange color) of *Escherichia coli* (PDB: 1SF2); (**b**) GABA-AT catalytic site cavity (in purple color) of *Pseudomonas* model. The cofactor (PLP) is represented as gray, blue, orange and red color Van der Waals spheres.

In Figure 5d,f,h, it is clearly observed that there is a hydrophobic region generated by the indoline aromatic ring. We assume that this ring generates unfavorable steric interactions with the GABA-AT enzyme. Figure 6 shows the cavity of the GABA-AT (PDB: 1SF2 from *Escherichia coli* and the model of the enzyme from *Pseudomonas*) [56]. The later has a Y-shape type, where one of its corners is surrounded by hydrophobic residues (where it would interact with the isobutyl and the aromatic ring) and the other two corners are surrounded by positively charged residues (with which the negatively charged atoms will interact). Therefore, using the “lock key” principle we can assume that the Y-shaped form of **18a** and **19a** analogues drastically favors their inhibitory activity.

#### 2.3.4. Molecular Similarity Analysis (MSA)

We performed MSA with respect to VGB **4** in order to explain why **18a** is relatively more active than **19a**. As these analogues were tested in vitro as racemic mixtures, both enantiomeric forms, (*R*) and (*S*) were considered.

**Figure 7:**
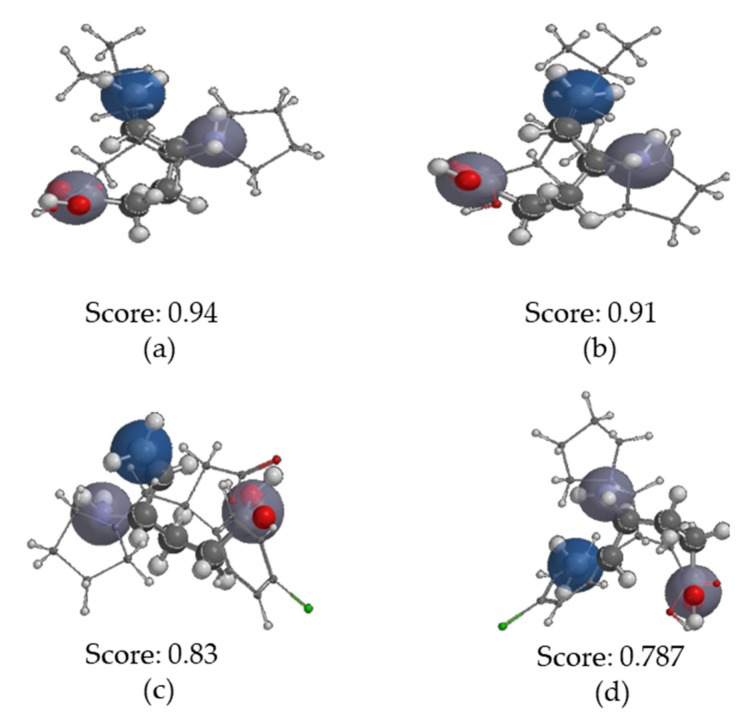
Structure alignment based on the CFD’s of VGB **4** in minimum energy conformation, with the active analogues. The spheres represent the CFDs. (**a**) (*S*)-**18a**; (**b**) (*R*)-**18a**; (**c**) (*S*)-**19a**; (**d**) (*R*)-**19a**.

**Figure 8:**
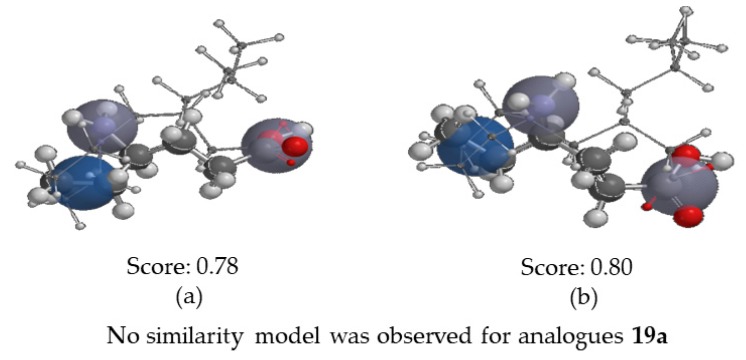
Structure alignment based on the CFD’s of VGB **4** in bioactive conformation with the active analogues. The spheres represent the CFD’s, previously described in Figure 3. (**a**) (*S*)-**18a**; (**b**) (*R*)-**18a**.

In Figure 7 and Figure 8 the MSA are displayed. The score reflects the quality of the superposition between the reference structure VGB **4** (minimum and bioactive conformations) and the synthesized analogues.

We observed that **18a**, whose GABA-AT inhibitory activity was 35.51%, has greater similarity with VGB **4**, both with the minimal energy conformation (Figure 7) and with the bioactive conformation (Figure 8). On the other hand, **19a**, whose GABA-AT inhibitory activity was 32.03%, showed less similarity with respect to the minimal energy conformation of VGB **4** and no similarity at all with its bioactive conformation. Based on these results, we suggest that **18a** has better activity than **19a**, since their interactions with GABA-AT are different, causing a decrease in their inhibitory activity. Compound **18a** has a greater flexibility than **19a**, caused by its isopropyl substituent, so that it can adopt a better conformation when interacting with GABA-AT. To confirm this assumption, we analyzed the molecular docking results.

### 2.4. Molecular Docking Calculations

#### 2.4.1. Molecular Docking over the Pseudomonas GABA-AT Model

**Figure 9:**
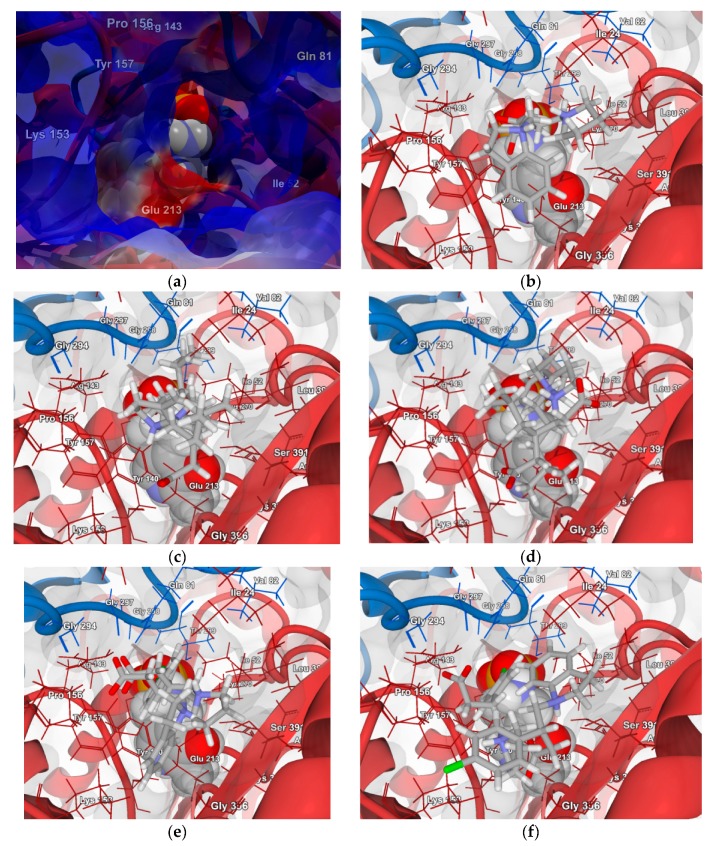
(**a**) Electrostatic potential map of *Pseudomonas* GABA-AT active site region. Blue, red and white colors represent regions with positive, negative and neutral electrostatic potential value, respectively; (**b**) GABA derivatives bound into the P*seudomonas* GABA-AT catalytic site; (**c**) (*S*)-Pregabalin analogues bound into the *Pseudomonas* GABA-AT catalytic site; (**d**) (*R*)-Pregabalin analogues bound into the *Pseudomonas* GABA-AT catalytic site; (**e**) (*S*)-Baclofen analogues bound into the *Pseudomonas* GABA-AT catalytic site; (**f**) (*R*)-Baclofen analogues bound into the *pseudomonas* GABA-AT catalytic site. PLP prosthetic group is shown as spacefill model.

To rationalize the GABA-AT inhibition shown by the GABA analogues, we carried out molecular docking studies over the *Pseudomonas* homology model. We analyzed the molecular docking calculations by means of the scaffold structure (GABA, Pregabalin **1** and Baclofen **2**). Figure 9 shows the molecular docking of all the GABA analogues over the *Pseudomonas* GABA-AT model and the electrostatic potential map of the active site; the blue, red and white colored surfaces represent positive, negative and neutral electrostatic potentials, access to the region where the PLP is located can be seen in the middle of the image. The detailed interactions of each molecule with *Pseudomonas* GABA-AT are shown in the supplementary data (Figures S20–S22); energy values of their interaction are shown in Table S1.

GABA analogues bind to the catalytic site of the *Pseudomonas* GABA-AT through their carboxylic group which interacts near the PLP molecule. This may be related to their molecular similarity with GABA. Nevertheless, due to their lack of a primary amine group and small size they do not interact with the GABA-AT enzyme. On the contrary, **9a** forms four strong HBs: two with Arg143, one with Tyr140 and one with Tyr157; **9b** forms three HBs: two with Arg143 and one with Tyr140. The planarity and size of the indoline ring does not allow the molecule to go deeper into the catalytic site.

**Figure 10:**
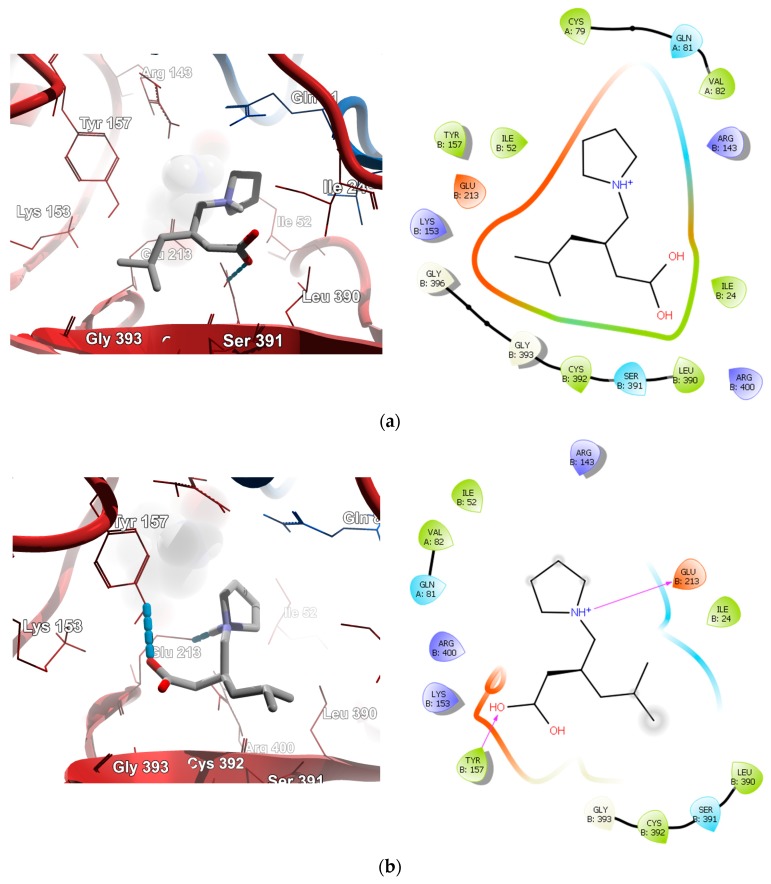
(**a**) (*R*)-**18a** hydrogen bond interactions (blue dashed lines) with *Pseudomonas* GABA-AT; (**b**) (*S*)-**18a** hydrogen bond interactions (blue dashed lines) with P*seudomonas* GABA-AT. The PLP prosthetic group is shown as spacefill model in the back of the image. The images were made with Molegro programs.

Pregabalin derivatives **18a** and **20b** do not show a specific pattern in their interaction with *Pseudomonas* GABA-AT. This may be due to their increased number of rotable bonds and that GABA-AT is not its biological target. All (*S*)-enantiomeric forms of Pregabalin analogues have a better interaction energy than the corresponding (*R*)-enantiomeric forms. From the experimental results, only compound **18a** exhibited inhibitory activity over *Pseudomonas* GABA-AT, the docking results shows that (*S*)-**18a** possesses a higher interaction value than (*R*)-**18a**. The shape and size of **18a** was crucial for its inhibitory activity, as its Y-type form allows **18a** to block the entry of GABA into the active site of the enzyme. As shown in Figure 10, the smaller size of the pyrrolidine ring, compared to indoline ring, allows the interaction of one part of the molecule with PLP.

Also, **18a** forms a stronger HB with its amine group and Glu213 than **20b** amine group and Tyr157. This fact is related to the highest partial charge negative value of the nitrogen atom in **18a** than in **20b** (strongest HBD above explained).

**Figure 11:**
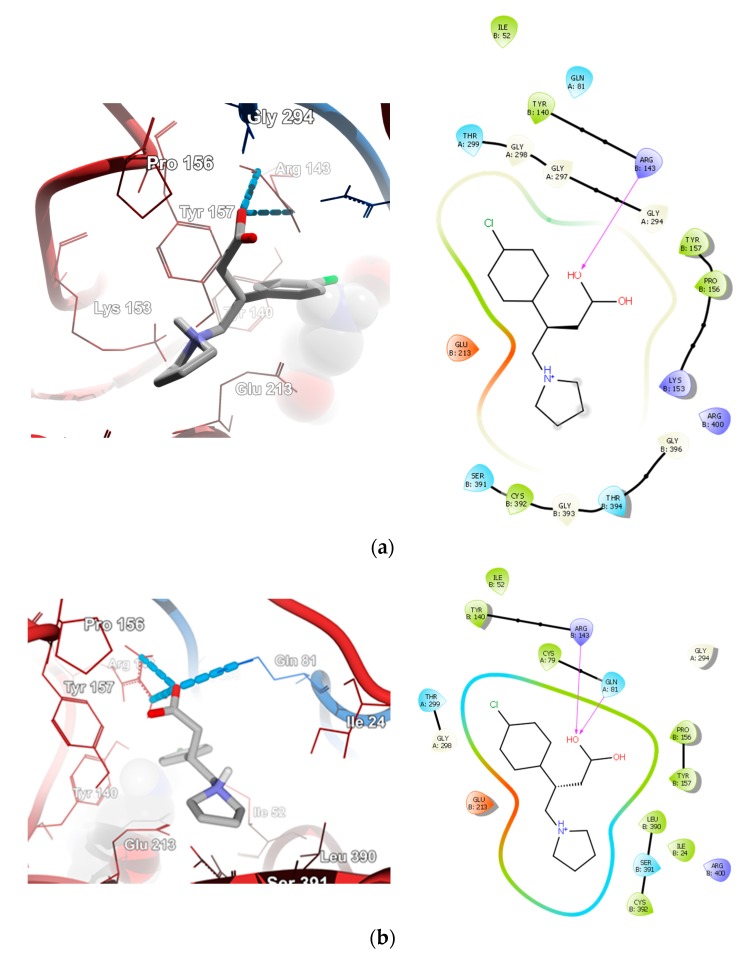
(**a**) (*R*)-**19a** hydrogen bond interactions (blue dashed lines) with *Pseudomonas* GABA-AT; (**b**) (*S*)-**19a** hydrogen bond interactions (blue dashed lines) with *Pseudomonas* GABA-AT. The PLP prosthetic group is shown as spacefill model. The images were made with Molegro programs.

Baclofen analogues **19a** and **21b** showed a similar form of interaction with *Pseudomonas* GABA-AT, where its chlorine atom points toward the prosthetic group. In the case of the Pregabalin analogues, the (*S*)-enantiomer presented higher negative values of interaction energy with *Pseudomonas* GABA-AT (that is, more stable complexes). As shown in Figure 11, (*S*)-**19a** displayed three strong HBs: two with Arg143 (side chain) and one with Gln81 (main chain); (*S*)-**21b** also displayed three strong HBs with the same residues as (*S*)-**19a**. Nevertheless, **21b** did not show inhibitory activity over *Pseudomonas* GABA-AT. This may be related the indoline ring greater volume, this may be related to the volume of the indoline ring which generates steric hindrance with the GABA-AT catalytic site (Figure S8). In addition, **19a** has a greater value of Ligand Efficiency (LE) than **21b** (see Table S1). LE reports on the interaction energy contributed by each heavy atom in the molecule, referred to the total interaction energy between ligand and GABA-AT.

From the docking results, we confirmed the importance of the shape and electronic distribution (partial charges over important atoms) of the GABA analogues as important factors to display inhibitory activity over *Pseudomonas* GABA-AT. A Y-type shape is related to the interaction with GABA-AT by means of the lock-key principle. In addition, the electronic distribution is related to the non-covalent interactions with GABA-AT, like the formation of strong HB, electrostatic and hydrophobic interactions.

**Figure 12:**
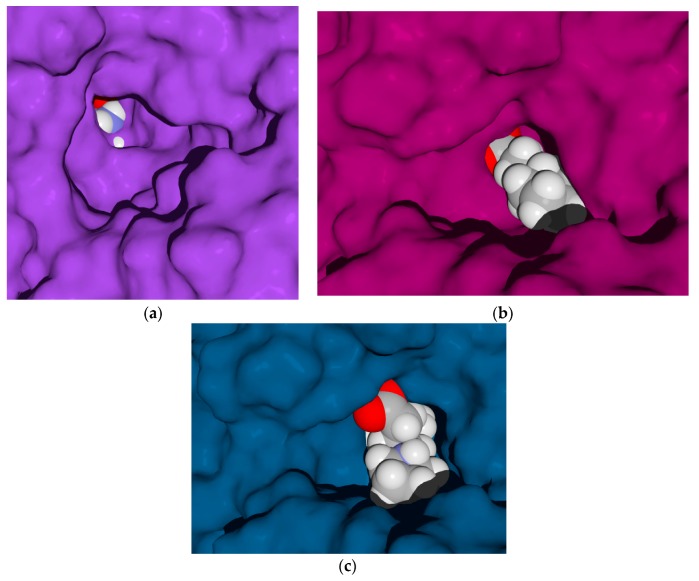
(**a**) Molecular surface of the catalytic site of *Pseudomonas* GABA-AT (purple color). PLP prosthetic group is shown as a spacefill model; (**b**) Molecular surface of *Pseudomonas* GABA-AT (pink color) interacting with **9a** (spacefill model); (**c**) Molecular surface of *Pseudomonas* GABA-AT (blue color) interacting with **19a** (spacefill model).

To explain the behavior of **9a** and **9b**, we analyzed the inductive effect caused by these compounds over the GABA-AT catalytic site (Figure 12). As it can be seen in Figure 12, in the absence of all ligands, the GABA-AT catalytic site shows a closed funnel shape (Figure 12a). However, when each analogue (compounds **9a** and **19a**) are introduced, an access path is opened to the catalytic site (Figure 12b,c). This path may enhance the activation of the GABA-AT enzyme, by facilitating the entry of the GABA molecule to the catalytic site. The small size and lower interaction energies of **9a** and **9b** with the enzyme, does not allow them to inhibit the activity of GABA-AT (Figure 12b). On the other hand, compounds **18a** and **19a** with a Y shape like form fulfill all the cavity, showing inhibitory activity. This can be related to their increased interaction energy (Table S2). All these factors together, allow **18a** and **19a** to block the entrance of the GABA molecule despite the path generated by the interaction of 1**9a** (Figure 12c).

**Figure 13:**
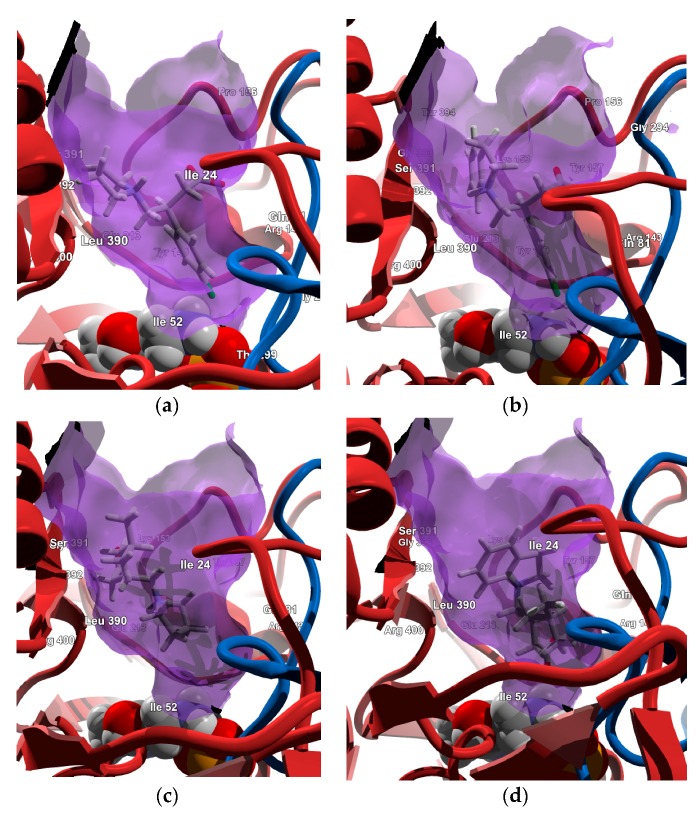
(**a**) (*S*)-**19a** interaction with *Pseudomonas* GABA-AT; (**b**) (*S*)-**21b** interaction with *Pseudomonas* GABA-AT; (**c**) (*S*)-**18a** interaction with *Pseudomonas* GABA-AT; (**d**) (*S*)-**20b** interaction with *Pseudomonas* GABA-AT. The PLP prosthetic group is shown as spacefill model. Molecular surface of the interaction between each compound and GABA-AT catalytic site (purple color).

To evaluate the effect of the indoline ring greater volume in the insertion to the catalytic site, we compared the interaction of **19a** and **21b** with GABA-AT (Figure 13a,b), with those of **18a** and **20b** (Figure 13c,d).

In Figure 13, it can be seen how the size of the indoline ring hinders the insertion of **20b** and **21b** to the catalytic site. The interaction of **20b** and **21b** occurs when the indoline ring is positioned at the top of the catalytic site (Figure 13b,d); this orientation may be due to its greater volume and more rigid structure. Analysis of the interactions of **19a** and **18a** on these figures shows that these compounds fit better in the catalytic site even if the pyrrolidine ring of **18a** interacts directly with PLP. In Figure 13 it can be appreciated how the indoline ring affects the insertion of the scaffold to the catalytic site. For compounds **20b** and **21b** their interaction occurs with the indoline ring in the top of the catalytic site (Figure 13b,d), this may be related to its greater volume and more rigidified structure. If the interaction of **19a** and **18a** is analyzed on these figures, we can observe they fit better in the catalytic site even if the pyrrolidine ring of **18a** interacts directly with PLP. This can be associated to its lower volume and greater flexibility between the isopropyl group and the pyrrolidine ring (Figure 13c).

Table 2 shows the values of the molecular volume descriptors related to the volume (AMR) and flexibility (RBF) of the molecules; where AMR accounts for the Molar Refractivity [57] and RBF for the rotable bond fraction of the molecules [58].

From Table 2 we can observe that insertion of the indoline ring increases the volume of the molecular scaffold (GABA, pregabalin and baclofen). In addition, this insertion reduces the rotable bond fraction of the molecules. Combining these results with the previous electronic analysis explained above, we propose that molecular shape (Y-type), flexibility, size and electronic distribution of the GABA analogues are factors that may be considered in order to improve their inhibitory activity over GABA-AT.

#### 2.4.2. Molecular Docking for Human GABA-AT Model

**Figure 14:**
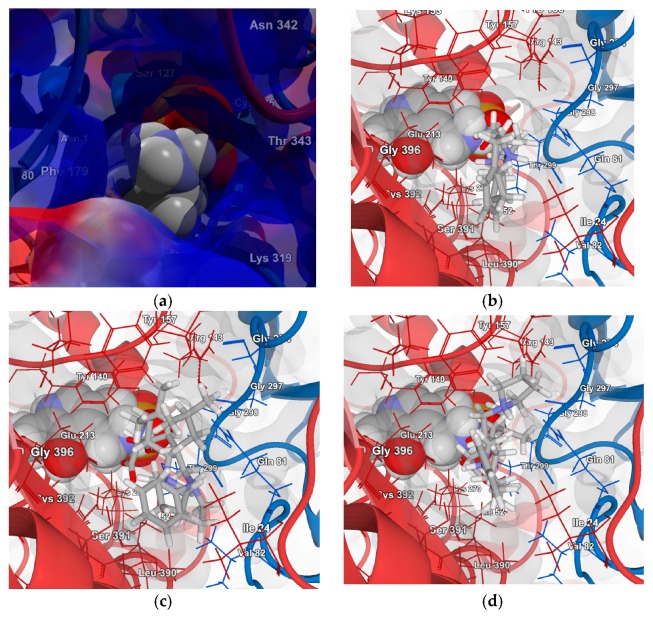

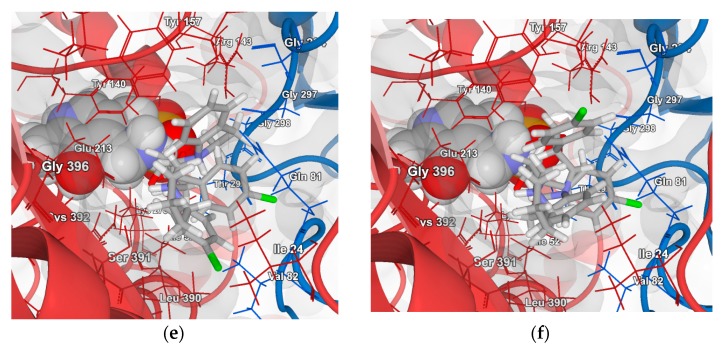
(**a**) Electrostatic potential map of human GABA-AT active site region. Blue, red and white colors represent regions with positive, negative and neutral electrostatic potential value, respectively; (**b**) GABA analogues bound into the human GABA-AT catalytic site; (**c**) (*S*)-Pregabalin analogues bound into the human GABA-AT catalytic site. (**d**) (*R*)-Pregabalin analogues bound into the human GABA-AT catalytic site. (**e**) (*S*)-Baclofen analogues bound into the human GABA-AT catalytic site. (**f**) (*R*)-Baclofen analogues bound into the human GABA-AT catalytic site. PLP prosthetic group and Fe_2_S_2_ cofactor are shown as spacefill models.

Since one of the main goals of this work is the identification of a compound that could be developed as a drug for clinical use, we carried out molecular docking calculations over a human GABA-AT model of all GABA analogues in order to study their interaction with the enzyme. In Figure 14 the binding mode of all the GABA analogues over the human GABA-AT model are shown. As for the *Pseudomonas* model, the catalytic site in human GABA-AT has primarily a positive electrostatic potential (blue colored surface) region and an entrance where PLP is located. The detailed interactions of all molecules with human GABA-AT are displayed in the supplementary data (Figures S23–S25), and the energy values of their interaction are shown in Table S2.

**Figure 15:**
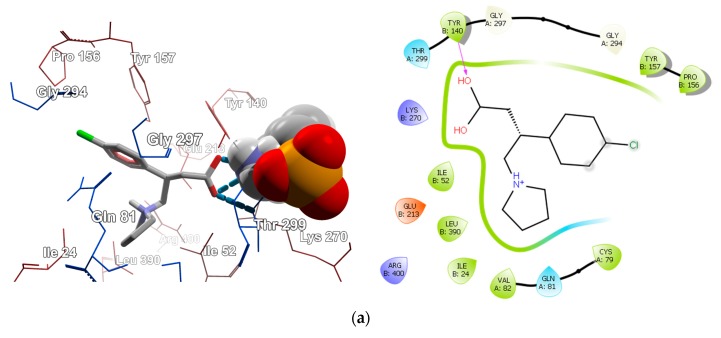

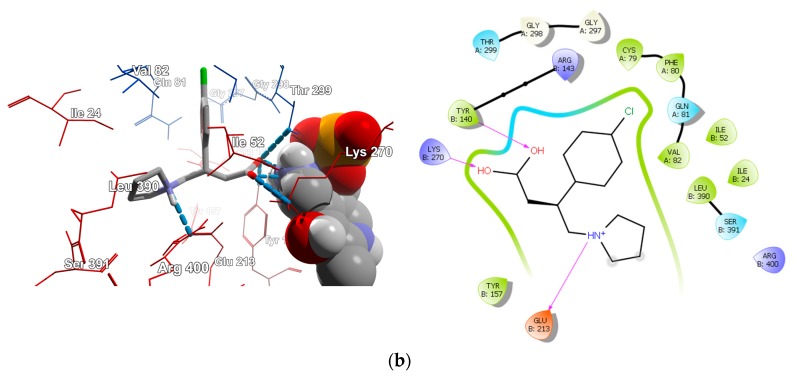
(**a**) (*R*)-**19a** hydrogen bond interactions (blue dashed lines) with human GABA-AT; (**b**) (*S*)-**19a** hydrogen bond interactions (blue dashed lines) with human GABA-AT. The PLP prosthetic group is shown as spacefill model. The images were made with Molegro programs.

The interaction between GABA analogues with human GABA-AT is similar to the one with *Pseudomonas* GABA-AT, where their carboxylic group is oriented toward the prosthetic group. Also, baclofen and pregabalin analogues bind in a similar way with human GABA-AT as with the *Pseudomonas* GABA-AT models. Pregabalin analogues do not show a clear pattern among them in their binding to human GABA-AT. On the other hand, baclofen analogues are oriented by the interaction of their carboxylic group with the prosthetic group, and the chloro-phenyl substituent points to the hydrophobic region composed by Ile24, Gln81, Val82, Gly297 and Gly298. From the interaction energy values of the docking results (Table S2), the best molecule to display possible enzyme inhibition activity is again **19a**. Figure 15 shows the binding interaction of **19a** with these amino acids.

The interaction of **19a** with human GABA-AT is much stronger with the (*S*)-enantiomer than with the (*R*)-enantiomer. The interaction of **19a** with human GABA-AT was much stronger with the (*S*)-enantiomer that with the (*R*)-enantiomer. (*S*)-**19a** forms three strong HBs: two by its carboxylic group with Tyr140 and Lys270, and one by its pyrrolidine group and Glu213.

## 3. Materials and Methods

### 3.1. Chemistry

All reagents and solvents were purchased from commercial suppliers and used without further purification. The reaction progress in the synthesis of all compounds was monitored on Merck aluminum plates precoated with silica gel 60 F254 (merck, KGaA, Dormstadt, Germany). Silica gel column chromatography was performed with Silica Gel 60G F254 (merck, KGaA, Dormstadt, Germany). Flasks, stirrer bars and hypodermic needles used for the generation of the Grignard and copper reagents were dried for ca. 12 h at 120 °C and allowed to cool in a dessicator over anhydrous calcium sulfate. Anhydrous solvents (ether and THF) were obtained by distillation from benzophenone ketyl. 1H NMR spectra were registered on a Varian INOVA 400 (400 MHz), 13C NMR (100 MHz). The spectra were recorded in D_2_O or CDCl_3_ solution, using TMS as the internal reference.

#### 3.1.1. General Procedure for the Preparation of **8a**–**c**

In a 25 mL round-bottomed flask, heterocycles **6a**–**c** (11.9 mmol) and ester **7** (11.9 mmol) were heated at 70 °C for 45 min with constant stirring. When the reaction was over, an aqueous solution of sodium bicarbonate was added until gas evolution ceased. The aqueous phase was extracted with ethyl acetate (3 × 10.0 mL) and the resulting organic layers were combined and dried over Na_2_SO_4_. The solvent was evaporated and the products purified by column chromatography (silica-gel; ethyl acetate-methanol 9:1) to afford the corresponding products.

*Methyl 4-(pyrrolidin-1-yl)butanoate* (**8a**). This compound was obtained as a light yellow oil, 0.74 g (62%). ^1^H-NMR (200 MHz, CDCl_3_) δ (ppm) = 3.59 (3H, s, OCH_3_), 2.42 (4H, m, H_pyrrolidine_), 2.38 (2H, t, *J* = 8.40 Hz, NCH_2_), 2.29 (2H, t, *J* = 7.6 Hz, CH_2_CO), 1.76 (2H, q, *J* = 7.2 Hz), 1.69 (4H, m, H_pyrrolidine_). ^13^C-NMR (50 MHz, CDCl_3_) δ (ppm) = 174.01 (1C, CO), 55.71 (2C, CH_2pyrrolidine_), 54.16 (1C, NCH_2_), 51.62 (1C, COOCH_3_), 32.28 (1C, CH_2_CO), 24.40 (2C, CH_2pyrrolidine_), 23.55 (1C, CH_2_CH_2_). HRMS-FAB *m*/*z* [M + H]^+^ calcd C_9_H_17_NO_2_: 171.1259; found (M + 1): 172.1248.

*Methyl 4-(indolin-1-yl)butanoate* (**8b**). This compound was obtained as a light yellow oil, 0.51 g (62%). ^1^H-NMR (200 MHz, CDCl_3_) δ (ppm) = 7.06 (1H, d, *J* = 7.4 Hz, H_aromatic_), 7.04 (1H, t, *J* = 7.4 Hz, H_aromatic_), 6.63 (1H, t, *J* = 7.2 Hz, H_aromatic_), 6.45 (1H, d, *J* = 8.4 Hz, H_aromatic_), 3.66 (3H, s, OCH_3_), 3.33 (2H, t, *J* = 8.0 Hz, CH_2indoline_), 3.09 (2H, t, *J* = 6.8 Hz, NCH_2_), 2.94 (2H, t, *J* = 8.2 Hz, CH_2indoline_), 2.43 (2H, t, *J* = 7.2 Hz, CH_2_CO), 1.93 (2H, q, *J* = 7.0 Hz, CH_2_CH_2_). ^13^C-NMR (50 MHz, CDCl_3_) δ (ppm) = 173.95 (1C, CO), 152.64 (1C, C_aromatic_), 130.00 (1C, C_aromatic_), 127.40 (1C, C_aromatic_), 124.52 (1C, C_aromatic_), 117.63 (1C, C_aromatic_), 106.99 (1C, C_aromatic_), 53.34 (1C, CH_2indoline_), 51.74 (1C, OCH_3_), 48.80 (1C, NCH_2_), 31.70 (1C, CH_2_CO), 28.79 (1C, CH_2indoline_), 23.15 (1C, CH_2_CH_2_). HRMS-FAB *m*/*z* [M + H]^+^ calcd C_13_H_17_NO_2_: 219.1259; found (M + 1): 220.1340.

*Methyl 4-(1H-imidazol-1-yl)butanoate* (**8c**). This compound was obtained as a light yellow oil, 0.55 g (68%). ^1^H-NMR (200 MHz, CDCl_3_) δ (ppm) = 7.43 (1H, s, H_imidazole_), 7.03 (1H, d, *J* = 1.05 Hz, H_imidazole_), 6.88 (1H, s, *J* = 1.25 Hz, H_imidazole_), 3.99 (2H, t, *J* = 6.95 Hz, NCH_2_), 3.65 (3H, s, OCH_3_), 2.27 (2H, t, *J* = 7.1 Hz, CH_2_CO), 2.07 (2H, q, *J*=7.05 Hz, CH_2_CH_2_). ^13^C-NMR (50 MHz, CDCl_3_) δ (ppm) = 172.94 (1C, CO), 137.25 (1C, C_imidazole_), 129.78 (1C, C_imidazole_), 118.82 (1C, C_imidazole_), 51.92 (1C, OCH_3_), 45.93 (1C, NCH_2_), 30.46 (1C, CH_2_CO), 26.30 (1C, CH_2_CH_2_). HRMS-FAB *m*/*z* [M + H]^+^ calcd C_8_H_12_N_2_O_2_: 168.0899; found (M + 1): 169.0988.

#### 3.1.2. General Procedure for the Preparation of **9a**–**c**

Esters **8a**–**c** (0.45 mmol) were dissolved in methanol (5.0 mL). An aqueous solution of lithium hydroxide (0.50 mmol) was added slowly and the resulting mixture was stirred for 24 h at 25 °C. When the reaction was over, the solvent was evaporated and the residue treated with a saturated aqueous solution of ammonium chloride. The aqueous layer was extracted with chloroform (3 × 10.0 mL), and then the organic layers were combined, dried with Na_2_SO_4_ and evaporated to give the corresponding products.

*(Pyrrolidin-1-yl)butanoic acid* (**9a**). This compound was obtained as a light yellow solid, 0.35 g (83%), mp 104–106 °C. ^1^H-NMR (200 MHz, CDCl_3_) δ (ppm) = 3.61 (2H, m, H_pyrrolidine_), 3.15 (2H, t, *J* = 7.60 Hz, NCH_2_), 3.02 (2H, m, H_pyrrolidine_), 2.43 (2H, t, *J* = 6.8 Hz, CH_2_CO), 2.05–1.93 (6H, m, H_pyrrolidine_, CH_2_CH_2_). ^13^C-NMR (50 MHz, CDCl_3_) δ (ppm) = 176.57 (1C, CO), 54.26 (2C, CH_2pyrrolidine_), 54.08 (1C, NCH_2_), 30.68 (1C, CH_2_CO), 22.86 (2C, CH_2pyrrolidine_), 20.92 (1C, CH_2_CH_2_). HRMS-FAB *m*/*z* [M + H]^+^ calcd C_8_H_15_NO_2_: 158.1103; found (M + 1): 158.1123.

*(Indolin-1-yl)butanoic acid* (**9b**). This compound was obtained as a light yellow oil, 0.45 g (76%). ^1^H-NMR (200 MHz, CDCl_3_) δ (ppm) = 10.70 (1H, s, OH), 7.06 (1H, d, *J* = 6.6 Hz, H_aromatic_), 7.04 (1H, t, *J* = 7.8 Hz, H_aromatic_), 6.64 (1H, t, *J* = 7.4 Hz, H_aromatic_), 6.47 (1H, d, *J* = 8.0 Hz, H_aromatic_), 3.31 (2H, t, *J* = 8.8 Hz, CH_2indoline_), 3.08 (2H, t, *J* = 7.0 Hz, NCH_2_), 2.97 (2H, t, *J* = 8.4 Hz, CH_2indoline_), 2.45 (2H, t, *J* = 7.4 Hz, CH_2_CO), 1.92 (2H, q, *J* = 7.0 Hz, CH_2_CH_2_). ^13^C-NMR (CDCl_3_, 50 MHz) δ (ppm) = 179.44 (1C, CO), 152.34 (1C, C_aromatic_), 130.12 (1C, C_aromatic_), 127.37 (1C, C_aromatic_), 124.49 (1C, C_aromatic_), 118.03 (1C, C_aromatic_), 107.36 (1C, C_arom_), 53.37 (1C, CH_2indoline_), 48.92 (1C, NCH_2_), 31.73 (1C, CH_2_CO), 28.73 (1C, CH_2indoline_), 22.76 (1C, CH_2_CH_2_). HRMS-FAB *m*/*z* [M + H]^+^ calcd C_12_H_15_NO_2_: 205.1103; found (M + 1): 206.1143.

*4-(1H-Imidazol-1-yl)butanoic acid* (**9c**). This compound was obtained as a light yellow solid, 0.36 g (83%), mp 109–111 °C. ^1^H-NMR (200 MHz, CDCl_3_) δ (ppm) = 8.78 (1H, s, H_imidazole_), 7.56 (1H, s, H_imidazole_), 7.49 (1H, s, H_imidazole_), 4.33 (2H, t, *J* = 7.20 Hz, NCH_2_), 2.47 (2H, t, *J* = 7.0 Hz, CH_2_CO), 2.22 (2H, q, *J* =7.0 Hz, CH_2_CH_2_). RMN ^13^C (50 MHz, D_2_O) δ (ppm) 177.05 (1C, CO), 134.71 (1C, C_imidazole_), 121.98 (1C, C_imidazole_), 119.98 (1C, C_imidazole_), 48.65 (1C, NCH_2_), 30.59 (1C, CH_2_CO), 25.01 (1C, CH_2_CH_2_). HRMS-FAB *m*/*z* [M + H]^+^ calcd C_7_H_10_N_2_O_2_: 154.0742; found (M + 1): 155.0731.

#### 3.1.3. General Procedure for the Preparation of **11a**, **11b**

In a 5.0 mL round-bottomed flask were mixed heterocycles **6a** or **6b** (5.2 mmol) and ester **10** (5.2 mmol). The mixture was stirred for 30 min at room temperature until the reaction was over. The resulting residue was dissolved in ethyl acetate and purified by column chromatography (silica gel, ethyl acetate) to afford the corresponding esters **11a** and **11b**.

*(E)-Ethyl 4-(pyrrolidin-1-yl)but-2-enoate* (**11a**). This compound was obtained as a light yellow oil, 0.50 g (54%). ^1^H-NMR (200 MHz, CDCl_3_) δ (ppm) = 6.99 (1H, dt, *J* = 10.2, 15.8 Hz, CH_2_CH_vinyl_), 5.98 (1H, dt, *J* = 1.4, 15.8 Hz, CH_vinyl_CO), 4.19 (2H, d, *J* = 7.2 Hz, OCH_2_), 3.25 (2H, dd, *J* = 1.8, 6.2 Hz, NCH_2_), 2.57–2.49 (m, 4H, CH_2pyrrolidine_), 1.87–1.75 (4H, m, CH_2pirrolidine_), 1.29 (3H, t, *J* = 7.0 Hz, CH_2_CH_3_). ^13^C-NMR (50 MHz, CDCl_3_) δ (ppm) = δ 166.38 (1C, CO), 145.82 (1C, CH_vinyl_), 122.61 (1C, CH_vinyl_), 60.38 (1C, OCH_2_), 56.95 (1C, NCH_2_), 54.31 (2C, CH_2pyrrolidine_), 23.73 (2C, CH_2pyrrolidine_), 14.39 (1C, CH_2_CH_3_). HRMS-FAB *m*/*z* [M + H]^+^ calcd C_10_H_17_NO_2_: 183.1259; found (M + 1): 184.1242.

*(E)-Ethyl 4-(indolin-1-yl) but-2-enoate* (**11b**). This compound was obtained as a light yellow oil, 0.83 g (86%). ^1^H-NMR (200 MHz, CDCl_3_) δ (ppm) = 7.15–7.01 (2H, m, H_aromatic_), 6.99 (1H, dt, *J* = 5.4, 15.8 Hz, CH_2_CH_vinyl_), 6.68 (1H, t, *J* = 7.8 Hz, H_aromatic_), 6.43 (1H, d, *J* = 8.0 Hz, H_aromatic_), 6.07 (1H, dt, *J* = 1.8, 15.8 Hz, CH_vinyl_CO), 4.18 (2H, d, *J* = 7.4 Hz, OCH_2_), 3.83 (2H, dd, *J* = 1.8, 5.4 Hz, NCH_2_), 3.37 (2H, t, *J* = 8.2 Hz, CH_2indolina_), 2.98 (2H, t, *J* = 8.2 Hz, CH_2indoline_), 1.28 (3H, t, *J* = 7.0 Hz, CH_2_CH_3_). ^13^C-NMR (50 MHz, CDCl_3_) δ (ppm) = 166.25 (1C, CO), 151.67 (1C, C_aromatic_), 144.34 (1C, CH_vinyl_), 130.03 (1C, C_aromatic_), 127.46 (1C, C_aromatic_), 124.64 (1C, C_aromatic_), 122.94 (1C, CH_vinyl_), 118.27 (1C, C_aromatic_), 107.27 (1C, C_aromatic_), 60.59 (1C, OCH_2_CH_3_), 53.89 (1C, CH_2indoline_), 50.62 (1C, NCH_2_), 28.82 (1C, CH_2indoline_), 14.48 (1C, CH_2_CH_3_). HRMS-FAB *m*/*z* [M + H]^+^ calcd C_14_H_17_NO_2_: 231.1259; found: 231.1247.

#### 3.1.4. General Procedure for the Conjugate Additions to **11a** and **11b**

In a 50 mL three-necked flask, CuI (2.2 mmol) was suspended in anhydrous ether at 0 °C under a nitrogen atmosphere. In a separate 50 mL round-bottomed flask, Mg turnings (4.4 mmol) were suspended in ether at room temperature. To the latter flask, 1-bromo-2-methylpropane or bromochlorobenzene (4.3 mmol) were added dropwise until the corresponding Grignard reagents were generated, as evidenced by the disappearance of the Mg turnings. Each Grignard solution was transferred via *cannula* to the CuI suspension at 0 °C to form the corresponding cuprate reagents **12** or **13**. Once formed, **11a** or **11b** dissolved in anhydrous ether were added slowly, maintaining the temperature at 0 °C. Each of the resulting mixtures was stirred for 30 min at 0 °C and then for 24 h at room temperature until the reaction was over. The reaction was quenched by the addition of 60.0 mL of an aqueous solution of ammonium chloride. The aqueous layer was then washed with CH_2_Cl_2_ (3 × 50.0 mL) and the organic layers combined and dried over Na_2_SO_4_. The solvent was evaporated and the residue purified by column chromatography (silica-gel, ethyl acetate) to give the corresponding products.

*Ethyl 5-methyl-3-(pyrrolidin-1-ylmethyl)hexanoate* (**14a**). This compound was obtained as a light yellow oil, 0.26 g (82%). ^1^H-NMR (200 MHz, CDCl_3_) δ (ppm) = 4.10 (2H, d, *J* = 7.4 Hz, OCH_2_), 2.54–2.13 (9H, m, CH_2pyrrolidine_, NCH_2_, CH_2_CH, CH_2_CO), 1.76–1.69 (4H, m, CH_2pyrrolidine_), 1.66 (1H, hept, *J* = 6.6 Hz, (CH_3_)_2_CH), 1.25 (3H, t, *J* = 7.0 Hz, CH_2_CH_3_), 1.18–1.06 (2H, m, CHCH_2_), 0.92 (3H, d, *J* = 2.2 Hz, CH_3_), 0.87 (3H, d, *J* = 2.4 Hz, CH_3_). ^13^C-NMR (50 MHz, CDCl_3_) δ (ppm) = 173.77 (1C, CO), 61.53 (1C, NCH_2_), 60.16 (1C, OCH_2_), 54.56 (2C, CH_2pyrrolidine_), 42.98 (1C, CHCH_2_), 39.10 (1C, CH_2_CO), 32.94 (1C, CH_2_CH), 25.55 (1C, (CH_3_)_2_CH), 23.85 (2C, CH_2pyrrolidine_), 23.15 (1C, CH_3_), 22.94 (1C, CH_3_), 14.54 (1C, CH_2_CH_3_). HRMS-FAB *m*/*z* [M + H]^+^ calcd C_14_H_27_NO_2_: 241.2042; found (M + 1): 242.2089.

*Ethyl 3-(4-chlorophenyl)-4-(pyrrolidin-1-yl)butanoate* (**15a**). This compound was obtained as a light yellow oil, 0.16 g (34%). ^1^H-NMR (200 MHz, CDCl_3_) δ (ppm) = 7.31–7.17 (4H, m, H_arom_), 4.00 (2H, d, *J* = 6.0 Hz, OCH_2_), 2.54–2.13 (9H, m, CH_2pyrrolidine_, NCH_2_, CH_2_CH, CH_2_CO), 1.76–1.69 (4H, m, CH_2pyrrolidine_), 1.11 (3H, t, *J* = 6.0 Hz, CH_2_CH_3_). ^13^C-NMR (50 MHz, CDCl_3_) δ (ppm) = 172.26 (1C, CO), 129.14 (2C, C_aromatic_), 128.92 (2C, C_aromatic_), 60.67 (1C, OCH_2_), 61.73 (1C, NCH_2_), 54.62 (2C, CH_2pyrrolidine_), 40.12 (1C, CH_2_CO), 32.70 (1C, CH_2_CH), 23.79 (2C, CH_2pyrrolidine_), 14.30 (1C, CH_2_CH_3_). HRMS-FAB *m*/*z* [M + H]^+^ calcd C_16_H_22_NO_2_Cl: 295.1339; found (M + 1): 296.1304.

*Ethyl 3-(indolin-1-ylmethyl)-5-methylhexanoate* (**16b**). This compound was obtained as a light yellow oil, 0.28 g (75%). ^1^H-NMR (200 MHz, CDCl_3_) δ (ppm) = 7.07–7.00 (2H, m, H_aromatic_), 6.61 (1H, td, *J* = 1.0, 7.1 Hz, H_aromatic_), 6.44 (1H, d, *J* = 8.2 Hz, H_aromatic_), 4.03 (2H, d, *J* = 7.0 Hz, OCH_2_), 3.42 (1H, dd, *J* = 7.4, 8.6 Hz, NCH_2_), 3.24 (1H, dd, *J* = 8.4, 17.2 Hz, NCH_2_), 3.01–2.85 (4H, m, CH_2indoline_, CH_2_CO), 2.40–2.85 (3H, m, CH_2indoline_, CH_2_CH), 1.70 (1H, hept, *J* = 6.6 Hz, (CH_3_)_2_CH), 1.35–1.20 (2H, m, CHCH_2_), 1.55 (3H, t, *J* = 7.4 Hz, CH_2_CH_3_), 0.94 (3H, d, *J*=4.8 Hz, CH_3_), 0.91 (3H, d, *J* = 4.4 Hz, CH_3_). ^13^C-NMR (50 MHz, CDCl_3_) δ (ppm) = 173.23 (1C, CO), 153.10 (1C, C_aromatic_), 129.70 (1C, C_aromatic_), 127.37 (1C, C_aromatic_), 124.39 (1C, C_aromatic_), 117.45 (1C, C_aromatic_), 106.84 (1C, C_aromatic_), 60.25 (1C, OCH_2_), 55.19 (1C, NCH_2_), 54.47 (1C, CH_2indoline_), 42.40 (1C, CHCH_2_), 38.25 (1C, CH_2_CO), 32.97 (1C, CH_2_CH), 28.79 (1C, CH_2indoline_), 25.58 (1C, (CH_3_)_2_CH), 23.21 (1C, CH_3_), 22.85 (1C, CH_3_), 14.33 (1C, CH_2_CH_3_). HRMS-FAB *m*/*z* [M + H]^+^ calcd C_18_H_27_NO_2_: 289.2042; found (M + 1): 290.2030.

*Ethyl 3-(4-chlorophenyl)-4-(indolin-1-yl)butanoate* (**17b**). This compound was obtained as a light yellow oil, 0.42g (57%). ^1^H-NMR (200 MHz. CDCl_3_) δ (ppm) = 7.31–7.17 (4H, m, H_aromatic_), 7.08–7.01 (2H, m, H_aromatic_), 6.63 (1H, td, *J* = 2.0, 8.0Hz, H_aromatic_), 6.43 (1H, d, *J* = 8.0 Hz, H_aromatic_), 4.00 (2H, d, *J* = 6.0 Hz, OCH_2_), 3.50 (1H, q, *J* = 6.0 Hz, CH_2_CH), 3.40–3.05 (4H, m, NCH_2_, CH_2indoline_), 2.91 (2H, t, *J* = 8.0 Hz, CH_2indoline_), 2.84 (1H, dd, *J* = 8.0, 15.0 Hz, CH_2_CO), 2.59 (1H, dd, *J* = 8.0, 17.0 Hz, CH_2_CO), 1.11 (3H, t, *J* = 6.0 Hz, CH_2_CH_3_). ^13^C-NMR (CDCl_3_, 50 MHz) δ (ppm) = 172.25 (1C, CO), 152.59 (1C, C_arom_), 140.94 (1C, C_arom_), 132.79 (1C, C_aromatic_), 129.67 (1C, C_aromatic_), 129.14 (2C, C_aromatic_), 128.92 (2C, C_aromatic_), 127.51 (1C, C_aromatic_), 124.62 (1C, C_aromatic_), 117.79 (1C, C_aromatic_), 106.68 (1C, C_aromatic_), 60.67 (1C, OCH_2_), 56.25 (1C, NCH_2_), 54.38 (1C, CH_2indoline_), 41.12 (1C, CH_2_CO), 38.81 (1C, CH_2_CH), 28.79 (1C, CH_2indoline_), 14.30 (1C, CH_2_CH_3_). HRMS-FAB *m*/*z* [M + H]^+^ calcd C_20_H_22_NO_2_Cl: 343.1339; found (M + 1): 344.1378.

#### 3.1.5. General Procedure for the Synthesis of **18**–**21**

Esters **14**–**17** (0.45 mmol) were dissolved in methanol (5.0 mL). To each of the resulting mixtures, 5.0 mL of an aqueous solution of lithium hydroxide (0.50 mmol) were added, followed by continuous stirring for 24 h at room temperature. The solvent was then evaporated and the residue washed with a saturated aqueous solution of ammonium chloride. The aqueous layer was extracted with chloroform (3 × 25.0 mL), and the organic layers were combined and dried over Na_2_SO_4_ to afford the corresponding products.

*Methyl-3-(pyrrolidin-1-ylmethyl)hexanoic acid* (**18a**). This compound was obtained as a light yellow oil, 0.067 g (70%). ^1^H-NMR (200 MHz, CDCl_3_) δ (ppm) = 2.54–2.13 (9H, m, CH_2pyrrolidine_, NCH_2_, CH_2_CH, CH_2_CO), 1.76–1.69 (4H, m, CH_2pyrrolidine_), 1.66 (1H, hept, *J* = 6.6 Hz, (CH_3_)_2_CH), 1.18–1.06 (2H, m, CHCH_2_), 0.92 (3H, d, *J* = 2.2 Hz, CH_3_), 0.87 (3H, d, *J* = 2.4 Hz, CH_3_). ^13^C-NMR (50 MHz, CDCl_3_) δ (ppm) = 173.77 (1C, CO), 61.53 (1C, NCH_2_), 54.56 (2C, CH_2pyrrolidine_), 42.98 (1C, CHCH_2_), 39.10 (1C, CH_2_CO), 32.94 (1C, CH_2_CH), 25.55 (1C, (CH_3_)_2_CH), 23.85 (2C, CH_2pyrrolidine_), 23.15 (1C, CH_3_), 22.94 (1C, CH_3_). HRMS-FAB *m*/*z* [M + H]^+^ calcd C_12_H_23_NO_2_: 213.1729; found (M + 1): 214.1719.

*(4-Chlorophenyl)-4-(pyrrolidin-1-yl)butanoic acid* (**19a**). This compound was obtained as a light yellow oil, 0.73 g (56%). ^1^H-NMR (200 MHz, CDCl_3_) δ (ppm) = 7.31–7.17 (4H, m, H_aromatic_), 2.54–2.13 (9H, m, CH_2pyrrolidine_, NCH_2_, CH_2_CH, CH_2_CO), 1.76–1.69 (4H, m, CH_2pyrrolidine_). ^13^C-NMR (50 MHz, CDCl_3_) δ (ppm) = 172.26 (1C, CO), 129.14 (2C, C_arom_), 128.92 (2C, C_arom_), 61.73 (1C, NCH_2_), 54.62 (2C, CH_2pyrrolidine_), 40.12 (1C, CH_2_CO), 32.70 (1C, CH_2_CH), 23.79 (2C, CH_2pyrrolidine_). HRMS-FAB *m*/*z* [M + H]^+^ calcd C_14_H_18_NO_2_Cl: 267.1026; found (M + 1): 268.1041.

*3-(Indolin-1-ylmethyl)-5-methylhexanoic acid* (**20b**). This compound was obtained as a light yellow oil, 0.14 g (74%). ^1^H-NMR (200 MHz, CDCl) δ (ppm) = 10.02 (1H, br, COOH), 7.11–7.01 (2H, m, H_aromatic_), 6.63 (1H, td, *J* = 0.8, 7.4 Hz, H_aromatic_), 6.45 (1H, d, *J* = 8.0 Hz, H_aromatic_), 3.44 (1H, dd, *J* = 8.4, 16.5 Hz, NCH_2_), 3.24 (1H, dd, *J* = 8.0, 17.2 Hz, NCH_2_), 2.97–2.84 (4H, m, CH_2indoline_, CH_2_CO), 2.25–2.46 (3H, m, CH_2indoline_, CH_2_CH), 1.70 (1H, hept, *J* = 6.6 Hz, (CH_3_)_2_CH), 1.17–1.30 (2H, m, CHCH_2_), 0.94 (3H, d, *J* = 4.0 Hz, CH_3_), 0.90 (3H, d, *J* = 4.0 Hz, CH_3_). ^13^C-NMR (50 MHz, CDCl_3_) δ (ppm) = 179.59 (1C, CO), 152.98 (1C, C_aromatic_), 129.94 (1C, C_aromatic_), 127.46 (1C, C_arom_), 124.52 (1C, C_arom_), 117.91 (1C, C_aromatic_), 107.18 (1C, C_aromatic_), 55.34 (1C, NCH_2_), 54.68 (1C, CH_2indoline_), 42.43 (1C, CHCH_2_), 38.13 (1C, CH_2_CO), 32.82 (1C, CH_2_CH), 28.85 (1C, CH_2indoline_), 25.61 (1C, (CH_3_)_2_CH), 23.18 (1C, CH_3_), 22.91 (1C, CH_3_). HRMS-FAB *m*/*z* [M + H]^+^ calcd C_16_H_23_NO_2_: 261.1729; found (M + 1): 262.1794.

*3-(4-Chlorophenyl)-4-(indolin-1-yl)butanoic acid* (**21b**).This compound was obtained as a light yellow oil, 0.46 g (96%). ^1^H-NMR (200 MHz CDCl_3_) δ (ppm) = 10.14 (1H, br, COOH), 7.24–7.01 (6H, m, H_aromatic_), 6.63 (1H, t, *J* = 8.0Hz, H_aromatic_), 6.39 (1H, d, *J* = 8.0 Hz, H_aromatic_), 3.40–2.96 (5H, m, CH_2_CH, NCH_2_, CH_2indoline_), 2.85 (2H, t, *J* = 8.0 Hz, CH_2indoline_), 2.70 (1H, dd, *J* = 8.0, 17.0 Hz, CH_2_CO), 2.48 (1H, dd, *J* = 8.0, 16.0 Hz, CH_2_CO). ^13^C-NMR (50 MHz, CDCl_3_) δ (ppm) = 178.52 (1C, CO), 152.52 (1C, C_aromatic_), 140.88 (1C, C_aromatic_), 132.76 (1C, C_aromatic_), 129.73 (1C, C_aromatic_), 129.09 (2C, C_aromatic_), 128.91 (2C, C_aromatic_), 127.52 (1C, C_aromatic_), 124.66 (1C, C_aromatic_), 117.91 (1C, C_aromatic_), 106.71 (1C, C_aromatic_), 56.33 (1C, NCH_2_), 54.44 (1C, CH_2indoline_), 41.06 (1C, CH_2_CO), 39.21 (1C, CH_2_CH), 28.77 (1C, CH_2indoline_). HRMS-FAB *m*/*z* [M + H]^+^ calcd C_18_H_18_NO_2_Cl: 315.1026; found (M + 1): 316.1104.

### 3.2. Enzymatic Assays

#### Enzyme Inhibitory Tests

The enzymatic reactions were carried out at final concentrations of 0.2 mM of α-ketoglutarate, 1.25 mM of β-NADP and 0.0209 mg/mL of GABA-AT from *Pseudomonas fluorescens* in a 0.1 M potassium pyrophosphate buffer solution, pH = 8.6, at 25 °C for 30 min. Once the incubation period was over, the β-NADPH absorbance was measured at λ_max_ = 340 nm. The experiments were carried out at final equimolar concentrations of 0.8 mM for compounds **9a**–**c**, **18**–**21**, VGB **4** and VPNa **5** versus 0.8 mM of GABA.

### 3.3. Computational Studies

#### 3.3.1. Conformational Analysis and Geometric Optimization

The three-dimensional structures of the referenced drugs and analogues **9a**,**b**, **18a**, **19b**, **20b** and **21b** were constructed in the Spartan program version 08 [59]. The equilibrium conformer was calculated by analyzing 10,000 conformers of each analogue using molecular mechanics with the SYBYL force field [60]. Subsequently, the equilibrium geometry was optimized at the semi-empirical level with the AM1 method [61], without restrictions of symmetry. To confirm that the structures of the molecules were at a minimum of the potential energy surface, the vibrational frequencies were calculated to ensure that all were positive.

#### 3.3.2. Determination of Natural Partial Charges and Electrostatic Potential Maps

To obtain values of electron density, a single point calculation was made on the equilibrium geometry structure of each molecule, using the density functional theory (DFT) with the B3LYP hybrid functional [62] and the 6-31G * [63] basis set. From these results, the natural partial charges type of all compounds under study were obtained, because the biological activity of compounds with functional groups such as carboxylic acids and amines is highly related to their ability to ionize at physiological pH. In addition, we calculated the molecular electrostatic potential (MEP) mapped over an electron density isosurface of 0.002 e^−^/Å^3^, which allows us to observe the shape of the molecule and the electron distribution on that surface. Regions with high electronic density (represented in red color) will possess negative values of electrostatic potential, and regions with low electronic density (represented in blue color) will possess positive values. This allows the identification of zones where nucleophilic (blue zones) or electrophilic (red zones) attacks are possible. Also, regions of the molecule with neutral charge (represented in green color) possess zero value of electrostatic potential (hydrophobic zones).

#### 3.3.3. Molecular Similarity Analysis

**Figure 16:**
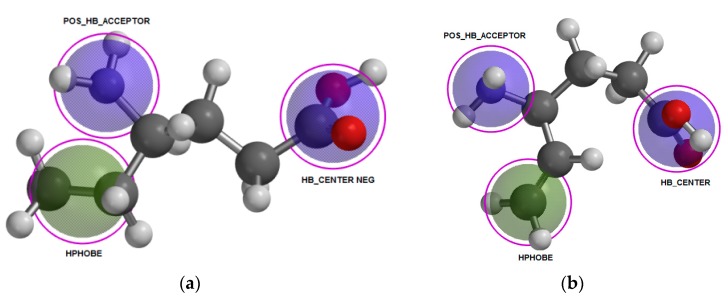
(**a**) Bioactive structure of VGB; (**b**) Minimum energy structure of VGB. The colored spheres are the CFD’s of VGB, where: HB_Center Neg is Hydrogen and Proton Negative Link Center, HPHOBE is Hydrophobic Region and POS_HB_Acceptor is Positive hydrogen bond acceptor center and basic region.

Molecular similarity analysis of all analogues were performed based on the chemical function descriptors (CFDs) of the VGB drug, which is an inhibitor of the GABA-AT enzyme. Since the bioactive conformation of a drug is not necessarily the minimum energy conformation, we carried out molecular similarity studies with two alignment models. In the first model, the minimal energy conformation of VGB was used, and in the second, the bioactive conformation of the VGB was employed. The bioactive conformation was extracted from the crystal structure of GABA-AT inactivated by VGB (PDB code: 1OHW) using the Spartan 08 program (Figure 16). In this structure, VGB is interacting with the PLP cofactor, therefore the characteristic double bond of VGB in the bioactive structure was non-existent, so it was necessary to modify such structure by adding the double bond with the Spartan 08 program.

#### 3.3.4. Homology Structural Modeling and Refinement

Given that a full 3D structure of the GABA aminotransferase of human and *P. fluorencens* is unavailable, we generated homology structural models. We used GABA aminotransferase structures available in the protein data bank server (PDB) as structural templates (Table 3 with the MODELLER 9.18 program [64]. According with the sequences alignment of GABA aminotransferases (Figure S1), those of bacteria are similar among them, and the same is true for animal GABA aminotransferases (human and wild boar).

For the model of human GABA aminotransferase we used the crystallographic structure 4y0h of *wild boar*, on account of its resolution (1.63 Å) and a higher sequence identity (~95%) to the human enzyme. The chains A and B were used to build the monomers. Once done, the dimer was assembled based in the same structure of 4y0h.

The model of *P. fluorencens* GABA-AT was performed using the 1sf2 crystallographic structure of *E. coli*. We used this structure instead of the *M. abscessus*, due a better conservation with respect of *E. coli* aminotransferase (~74% of identity). Again the chains A and B were used to build the monomers and the dimer was assembled according to the 1sf2 structure.

In the case of the bacterial GABA aminotransferases, both crystallographic structures of *Mycobacterium* and *E. coli* are a tetramers instead of a dimers as in animals; however the catalytic site is located in the interface of each dimer instead of tetramer interface, suggesting that dimer is the minimal catalytic form. This agrees with experimental studies suggesting that the dimer of bacteria is functional [56]; therefore, we used the bacterial dimer in this work.

Finally to optimize the complete structures of GABA-AT models, we used the CHARMM36 force field within CHARMM38b1 [65] to add hydrogen atoms and carry out a 100 step steepest decent minimization fixing all heavy atoms, followed by a conjugate gradient minimization of the full structure to remove bad contacts and clashes. These last protein conformations obtained were employed to carry out the molecular docking calculations.

To incorporate the prosthetic group (PLP) in the homology models an alignment employing a crystal structure that possessed the PLP was done (3r4t and 1ohw for the *Pseudomonas* and *human* models respectively). Additionally, from this alignment the Fe_2_/S_2_ (inorganic) cluster, present in the crystal structures of *Wild boar* as a cofactor, was added to the human GABA-AT model.

#### 3.3.5. Molecular Docking Calculations

Molecular docking calculations of all the GABA derivatives (including *R* & *S* enantiomers for molecules of series 2 and 3) over *Pseudomonas* and human GABA-AT structures (Figures S26 and S27 respectively), generated in the homology modeling process, were done in Molegro Virtual Docker (MVD) 6.0 software [66,67]. The cavities were detected by the expanded Van der Waals sphere method. The calculations were performed over the catalytic site with a cavity volume of 137.73 Å^3^ and 339.46 Å^3^ for the *Pseudomonas* and human model, respectively. The cavity selection was based on the results of the kinetic experiments, were it its shown that the GABA derivatives possess an uncompetitive inhibition mechanism. We employed the minimal geometries of all the GABA derivatives in their zwitterionic form, and the Mulliken partial charges obtained by the DFT calculations. The residues within a distance of 6 Å were set as flexible. A 2000 minimization steps for each flexible residue and 2000 steps of global minimization per run were set. The MolDock Optimizer search function, based on a evolutionary algorithm, was used. A total of 20 runs with a maximum of 4000 iterations using a population of 200 individuals (for the *Pseudomonas* model) and 400 individuals (for the *human* model) per run were set. To calculate the interaction energy, we used the scoring function Moldock Score [GRID]. The scoring function GRID was set at 0.2 Å and the search sphere fixed as 15 Å radius. For the energy analysis of the ligand, the electrostatic internal interactions, internal hydrogen bonds and the sp^2^-sp^2^ torsions were considered. The method was validated by reproducing the experimental binding mode of the reference inhibitor within the 3r4t crystal structure to validate the *Pseudomonas* model, and 1ohw and 1ohy to validate the *human* model, with a Root Media Square Deviation (RMSD) of 1.7 Å, 1.3 Å and 1.8 Å respectively (Figures S16–S18).

## 4. Conclusions

The synthesis of seven analogues of GABA, compounds **9a**–**c** and **18**–**21,** where the γ-nitrogen is part of a heterocyclic ring system, was accomplished by a short and efficient synthetic route. Attachment of these heterocyclic moieties to the GABA backbone, has the potential to make these compounds GABA-AT inhibitors as it occurs with compounds **18a** and **19a**. Regarding their capacity to inhibit GABA-AT, enzymatic inhibition tests showed compounds **18a** and **19a** as the most promising compounds on this series of analogues. Our computational results suggest that compound **19a** is a good candidate for future in vivo biological test in order to confirm its inhibitory biological activity over the human GABA-AT enzyme. These results open the possibility to explore new series of GABA analogues as reversible GABA-AT inhibitors, with a greater diversity of heterocyclic scaffolds.

## Figures and Tables

**Table 1:** Partial natural type charges of all molecules-(*S*)-enantiomers under study, according to the DFT analysis.

Natural Charges									
	VGB 4	1	2	9b	21b	20b	9a	19a	18a
Neutral N	−0.878	−0.923	−0.914	−0.523	−0.52	−0.521	−0.534	−0.548	−0.549
Neutral O	−0.731	−0.709	−0.741	−0.737	−0.736	−0.731	−0.716	−0.74	−0.735

**Table 2:** Values of the flexibility and molecular volume descriptors of the GABA analogues.

Molecule	RBF	AMR (${Å}^{3}$)
**9a**	0.148	40.969
**9b**	0.125	56.516
**18a**	0.154	59.191
**19a**	0.132	70.341
**20b**	0.136	74.738
**21b**	0.116	85.887

**Table 3:** GABA aminotransferase crystal structures available in PDB server.

PDB_ID	Resolution (Å)	Organism	% Identity Respect to Human	% Identity Respec to *P. fluorencens*
4zsw	1.70	*Wild boar*	95.46	27.52
4y0h	1.63	*Wild boar*	95.46	27.52
4y0i	1.66	*Wild boar*	95.46	27.52
1ohv	2.30	*Wild boar*	95.46	27.52
1ohw	2.30	*Wild boar*	95.46	27.52
1sf2	2.40	*E. coli*	26.35	73.82
4ffc	1.80	*M. abscessus*	27.99	42.79

## Supplementary Materials

The following are available online, Figures S1–S14: describe NMR data for all compound, Figures S17–S27: computational data, Tables S1 and S2 describe computational data.
