# Supplementary material for: Novel-Substituted Heterocyclic GABA Analogues. Enzymatic Activity against the GABA-AT Enzyme from Pseudomonas fluorescens and In Silico Molecular Modeling
Source: Molecules. 2018 May 9;23(5):1128. doi: 10.3390/molecules23051128 (PMC6099672; doi:10.3390/molecules23051128)
Supplement: Supplementary file 1 [file molecules-23-01128-s001.pdf]

Supplementary Information

Erika Tovar-Gudiño[1], Juan Alberto Guevara-Salazar [2], José Raúl Bahena-Herrera [3], José Guadalupe Trujillo-Ferrara [2], Zuleyma Martínez-Campos [1], Rodrigo Said Razo-Hernández [3], Angel Zamudio [3], Nina Pastor [3], Mario Fernández-Zertuche [1*]

Figure S1	2
Figure S2	3
Figure S3	4
Figure S4	5
Figure S5	6
Figure S6	7
Figure S7	8
Figure 8S	9
Figure S9	10
Figure S10	11
Figure S11	12
Figure S12	13
Figure S13	14
Figure S14	15
Figure S15	16
Figure S16	17
Figure S17	17
Figure S18	18
Figure S19	19
Figure S20	20
Figure S21	21
Figure S22	22
Table S1	22
Figure S23	23
Figure S24	25
Figure S25	26
Table S2	26
Figure S26	27
Figure S27	28

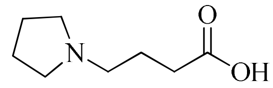

9a

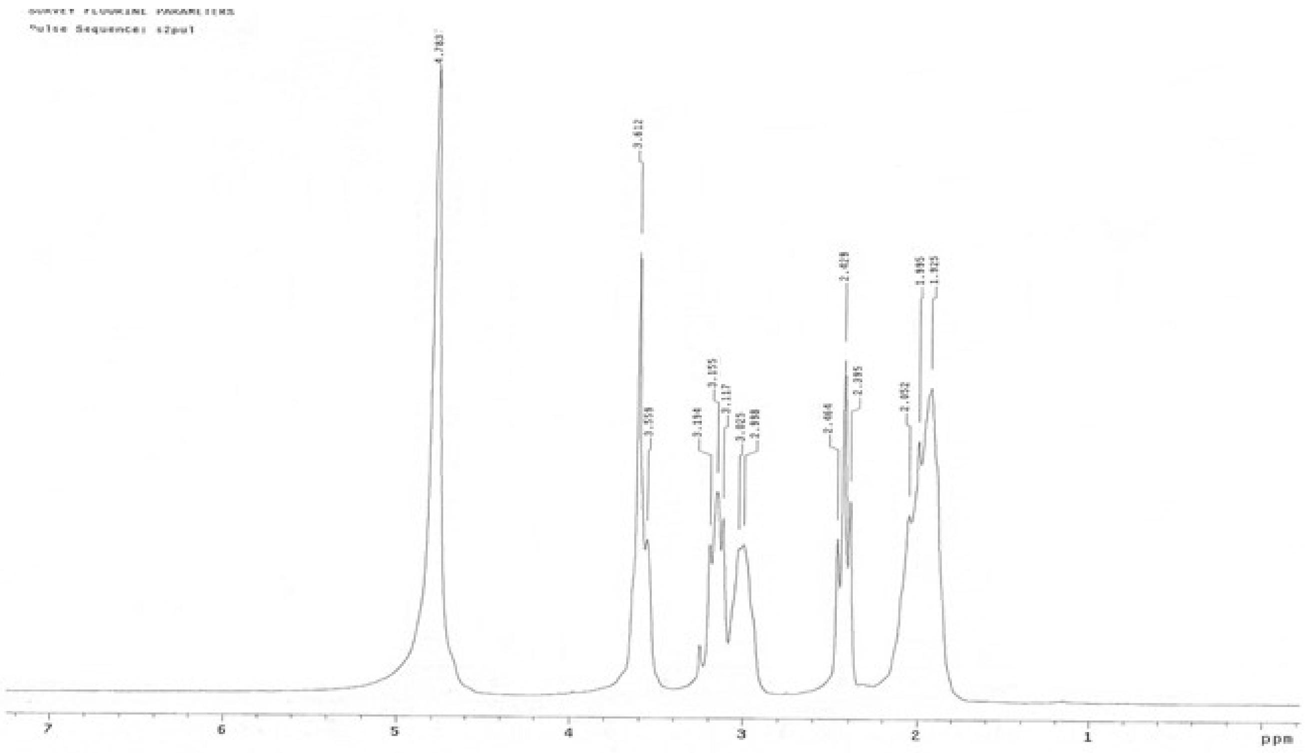

Figure S1. ^{1}H NMR (D$_2$O, 200 MHz) of **4-(Pyrrolidin-1-yl)butanoic acid (9a)**.

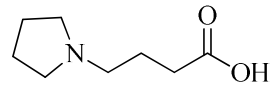

9a

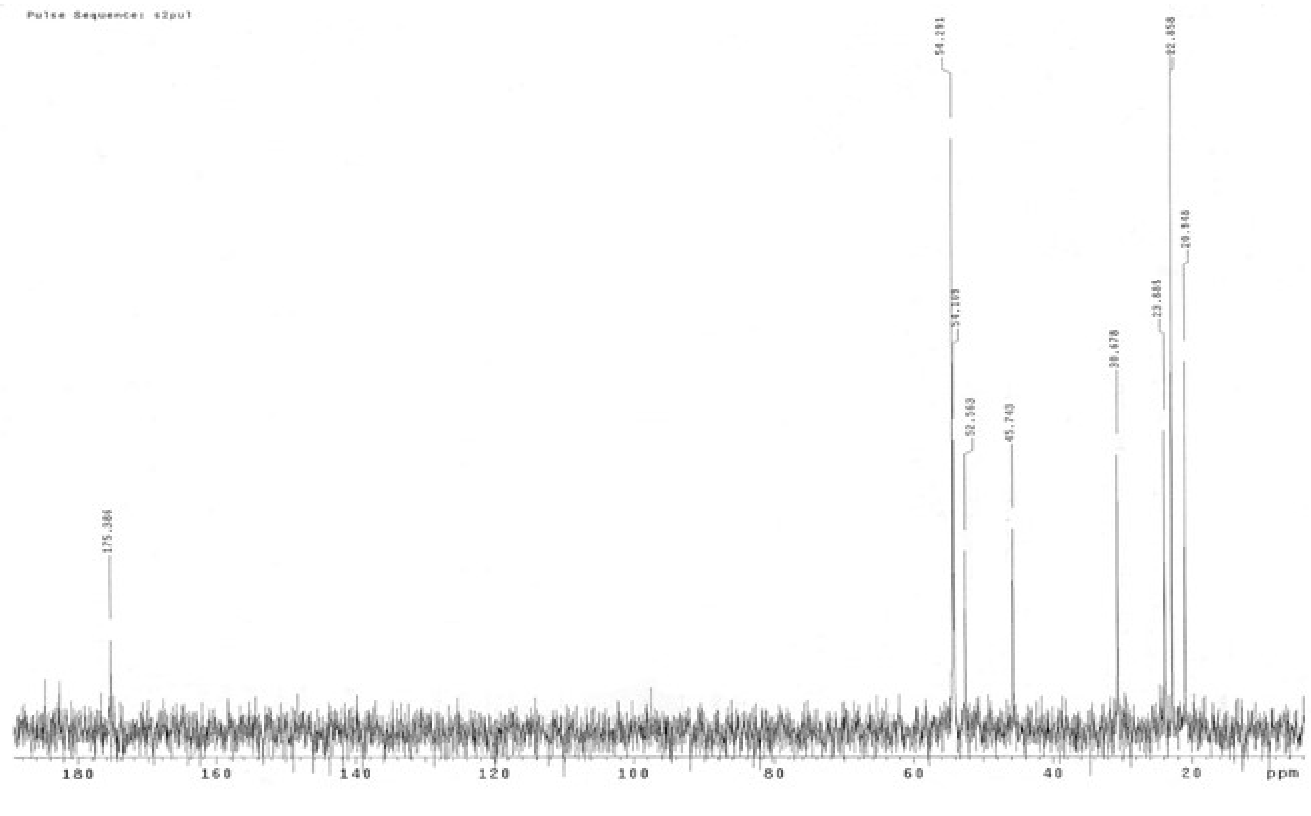

Figure S2. ^{13}C NMR (D$_2$O, 200 MHz) of **4-(Pyrrolidin-1-yl)butanoic acid (9a)**.

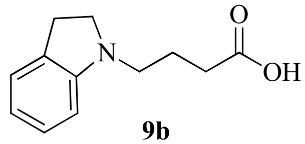

9b

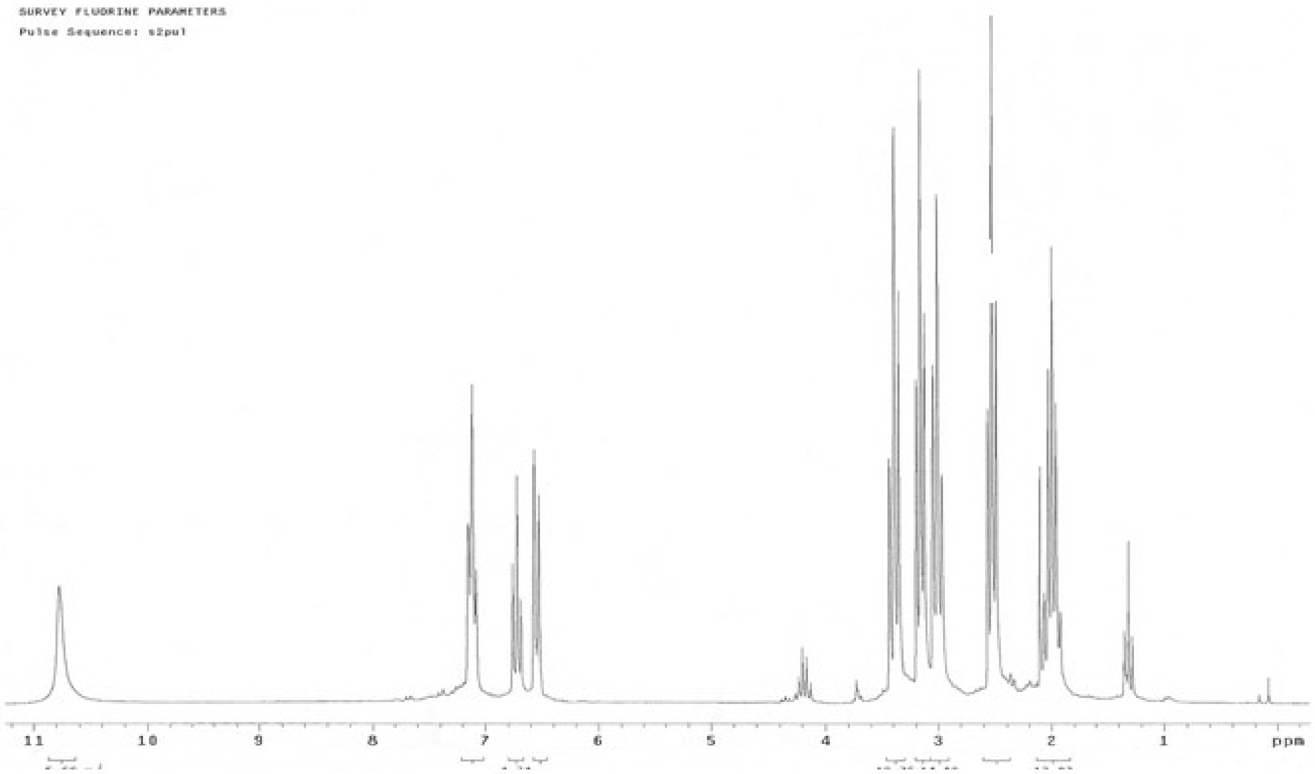

Figure S3. ^{1}H NMR (CDCl$_3$, 200 MHz) of **4-(Indolin-1-yl)butanoic acid (9b)**.

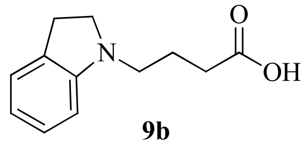

9b

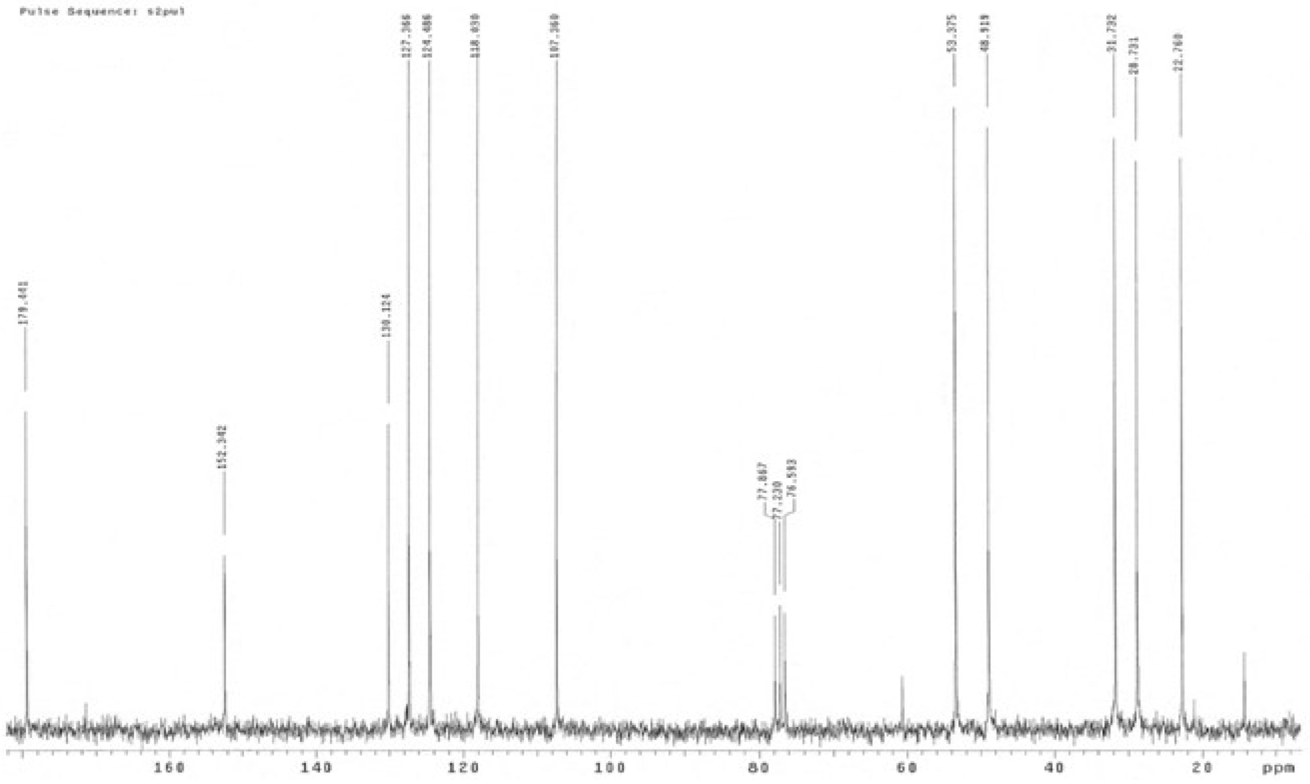

Figure S4. ^{13}C NMR ($CDCl_3$, 200 MHz) of **4-(Indolin-1-yl)butanoic acid (9b)**.

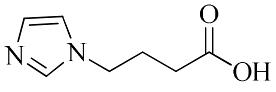

9c

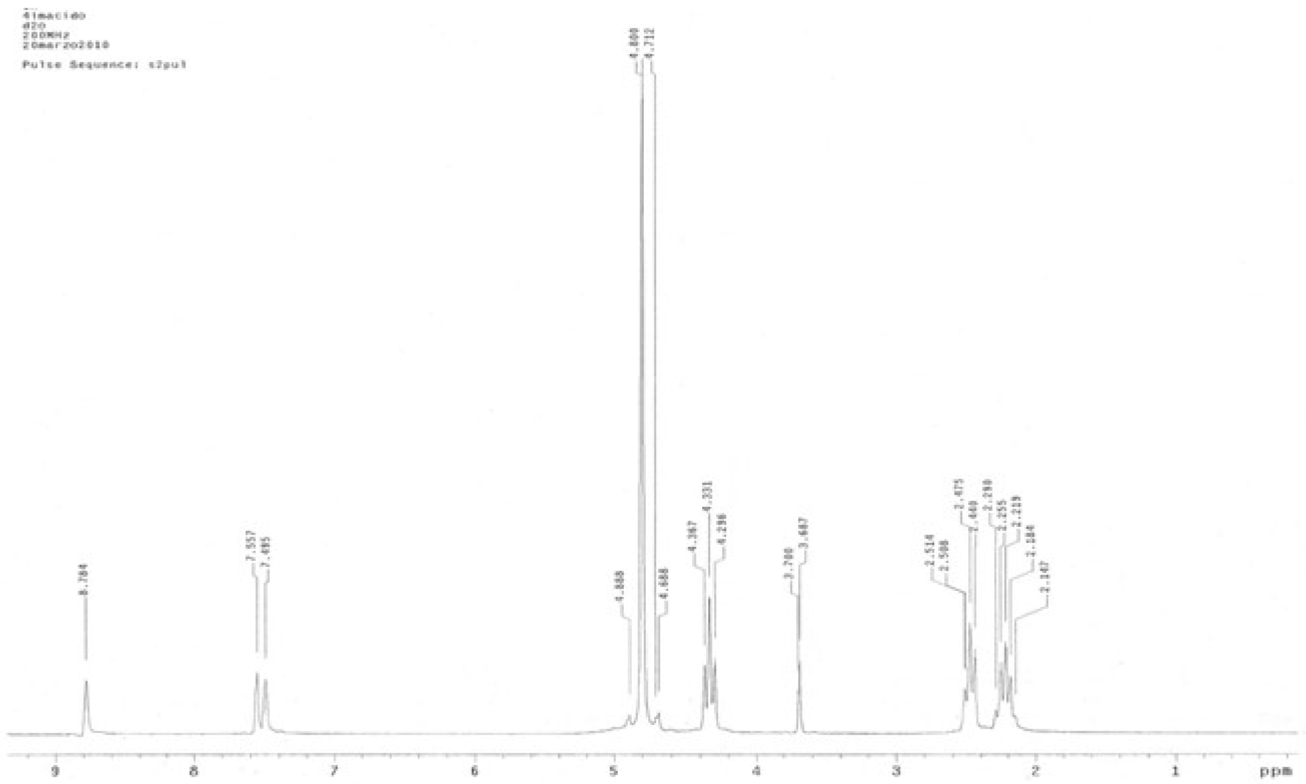

Figure S5. ^{1}H NMR (D_2O, 200 MHz) of **4-(1*H*-Imidazol-1-yl)butanoic acid (9c)**.

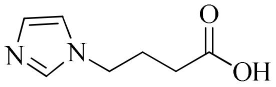

9c

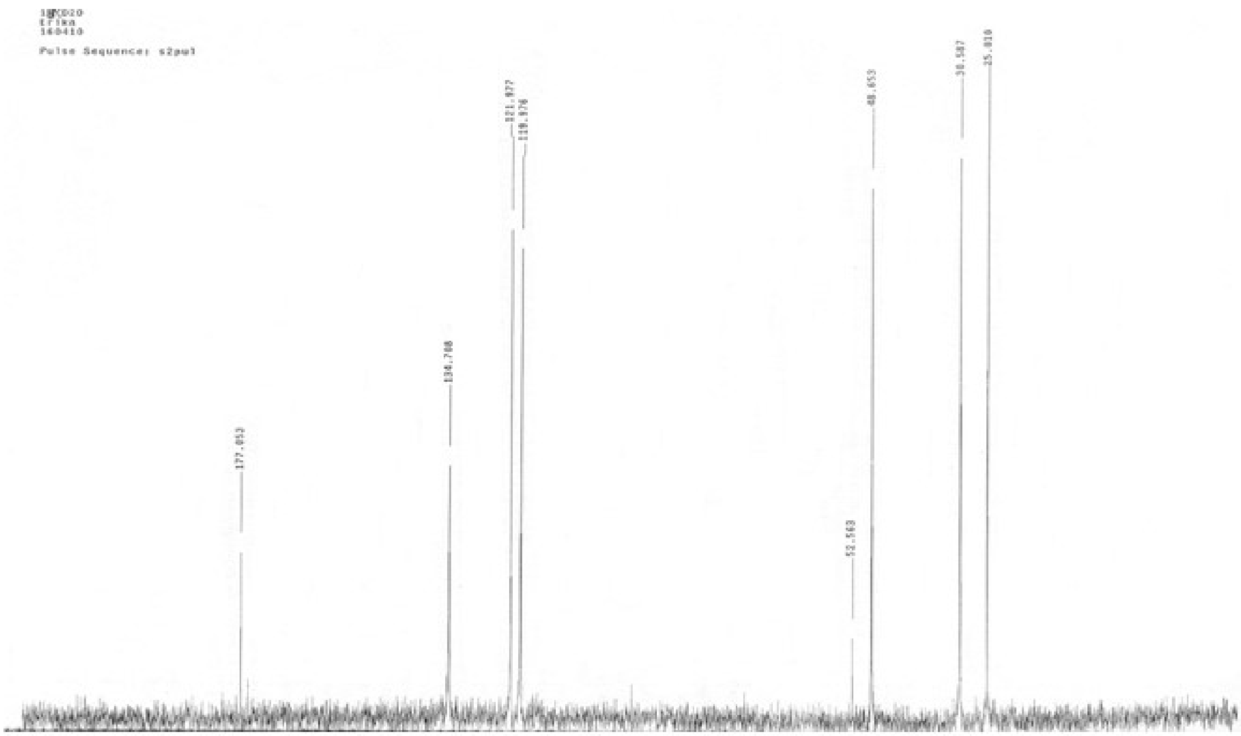

Figure S6. ${}^{13}C$ NMR (D$_2$O, 200 MHz) of **4-(1*H*-Imidazol-1-yl)butanoic acid (9c)**.

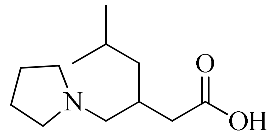

18a

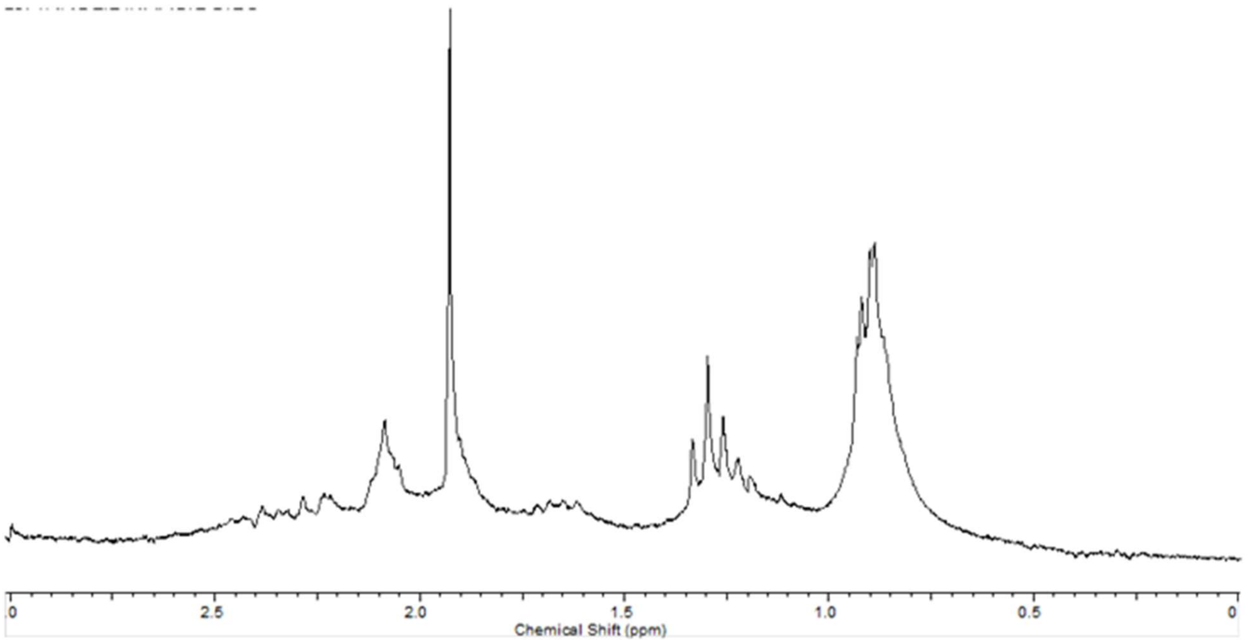

Figure S7. ^{1}H NMR (D_2O, 200 MHz) of **5-methyl-3-(pyrrolidin-1-ylmethyl)hexanoic acid (18a)**.

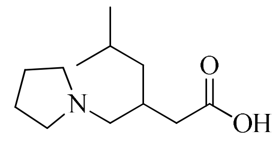

18a

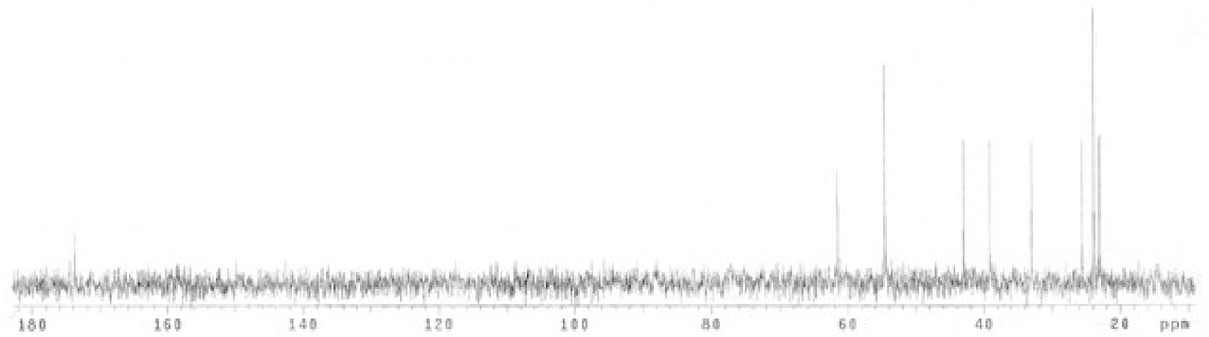

Figure S8. ^{13}C NMR (D$_2$O, 200 MHz) of **5-methyl-3-(pyrrolidin-1-ylmethyl)hexanoic acid (18a)**.

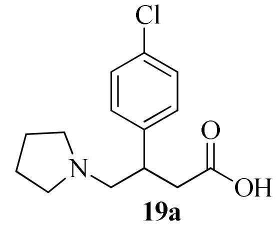

19a

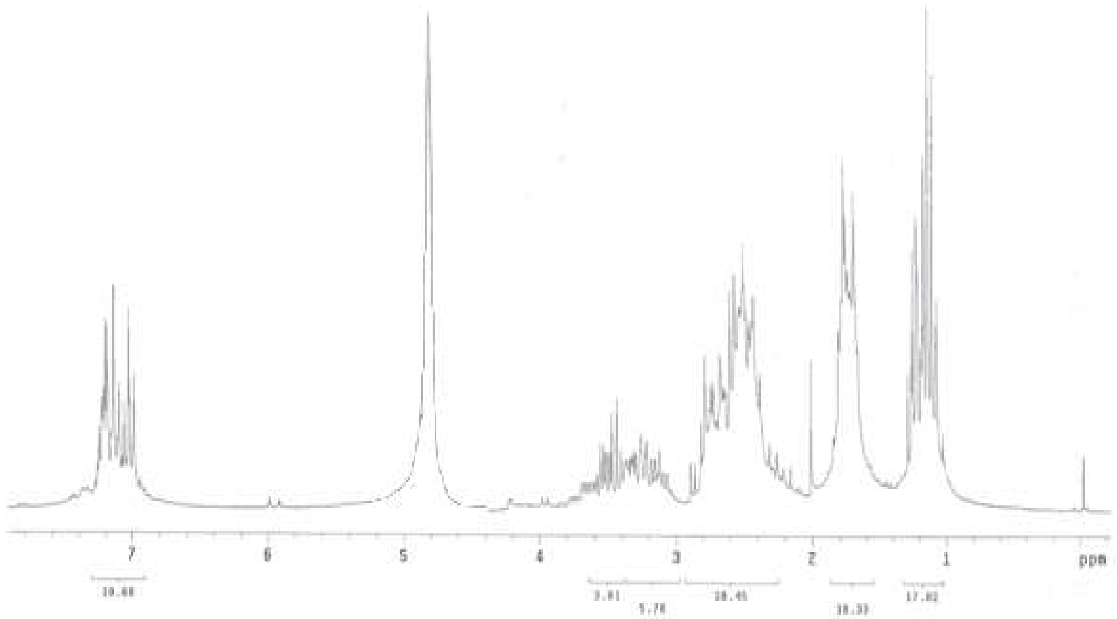

Figure S9. ^{1}H NMR (D_2O, 200 MHz) of **3-(4-chlorophenyl)-4-(pyrrolidin-1-yl)butanoic acid (19a)**.

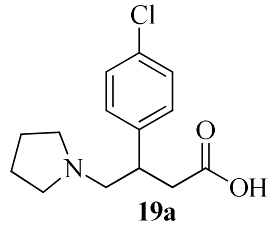

19a

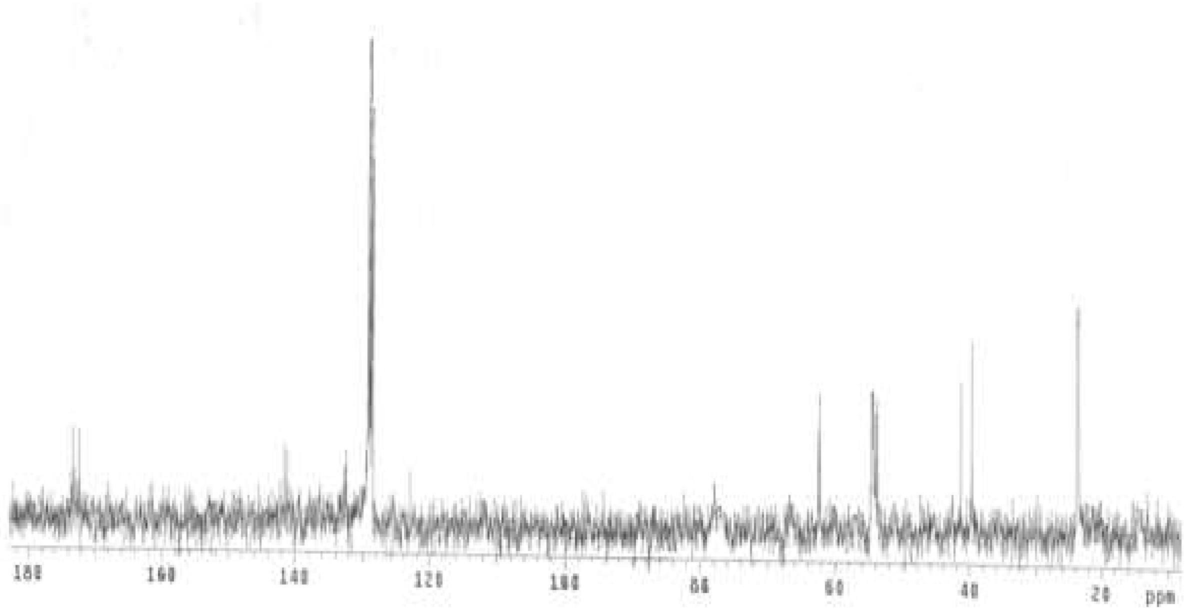

Figure S10. ^{13}C NMR (D_2O, 200 MHz) of **3-(4-chlorophenyl)-4-(pyrrolidin-1-yl)butanoic acid (19a)**.

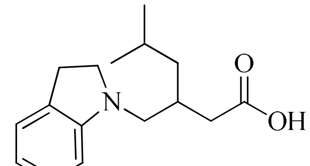

20b

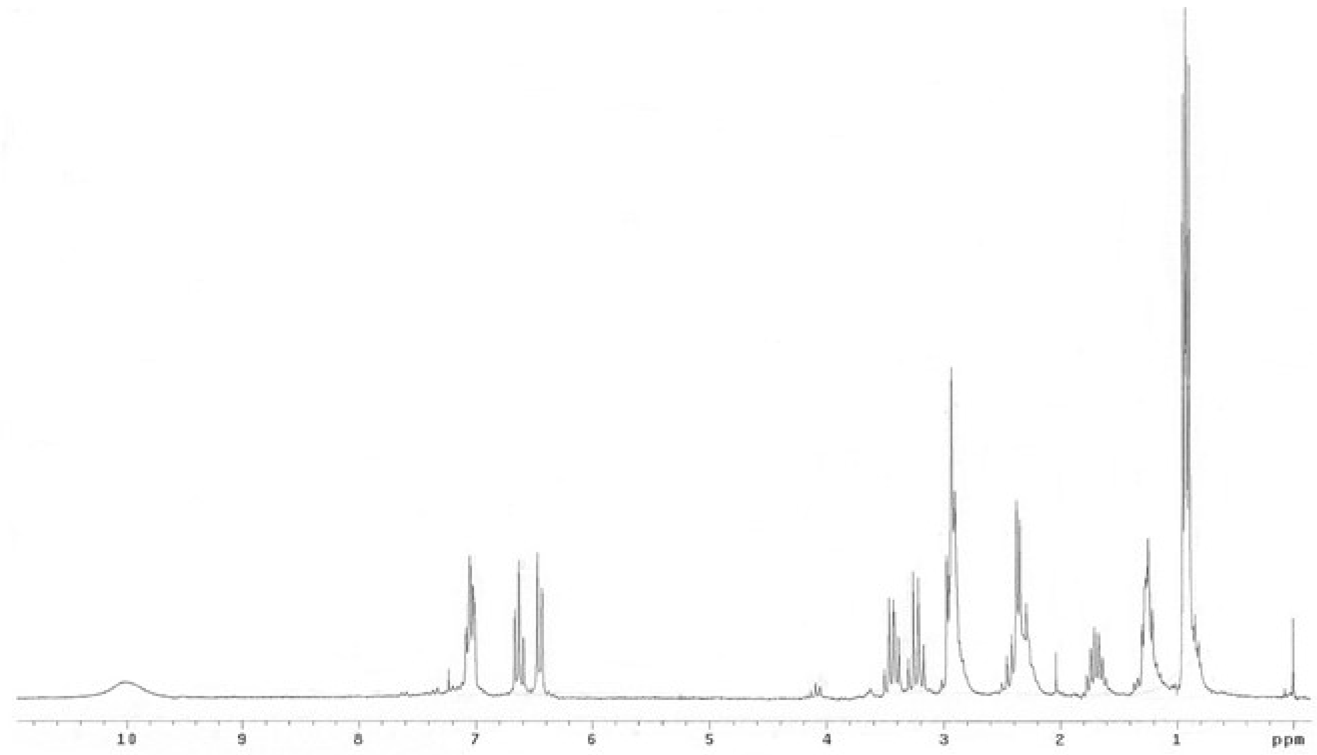

Figure S11. ^{1}H NMR (CDCl$_3$, 200 MHz) of **3-(Indolin-1-ylmethyl)-5-methylhexanoic acid (20b)**.

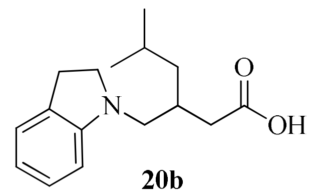

20b

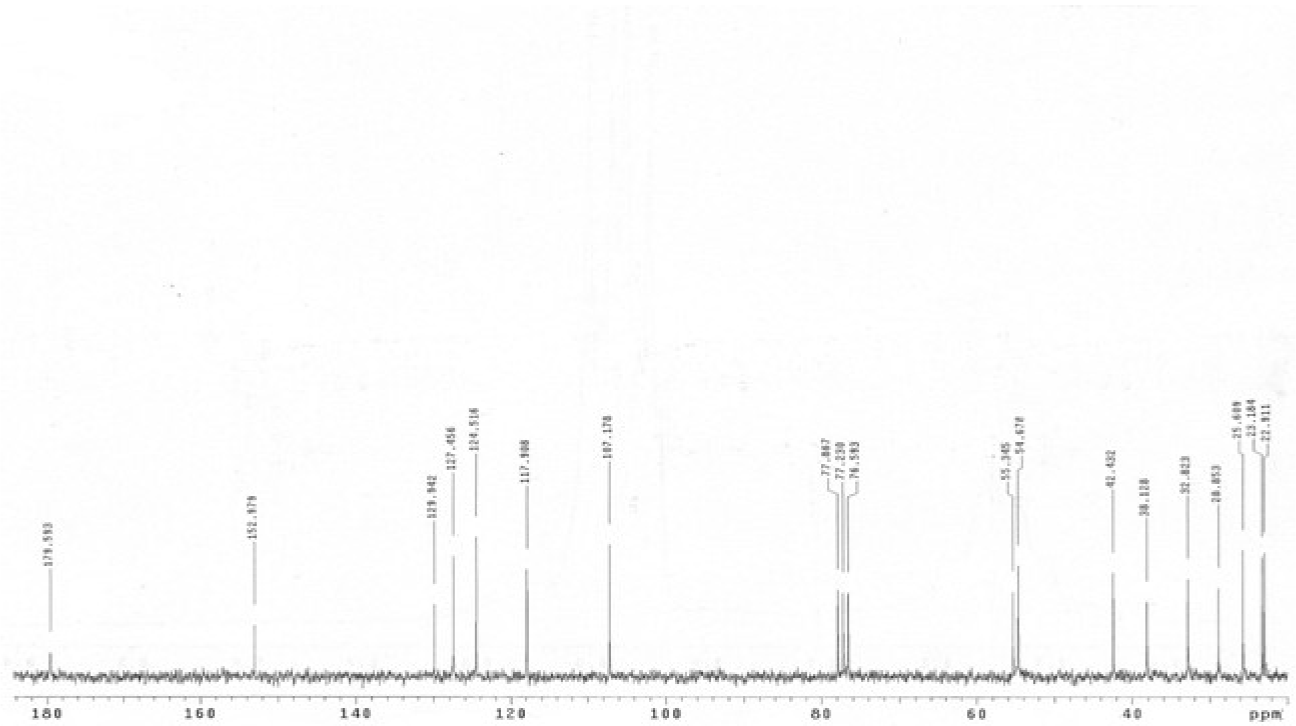

Figure S12. ^{13}C NMR (CDCl$_3$, 200 MHz) of **3-(Indolin-1-ylmethyl)-5-methylhexanoic acid (20b)**.

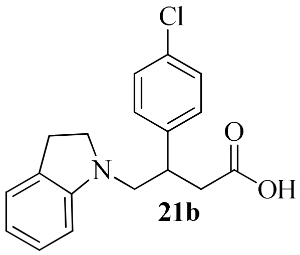

21b

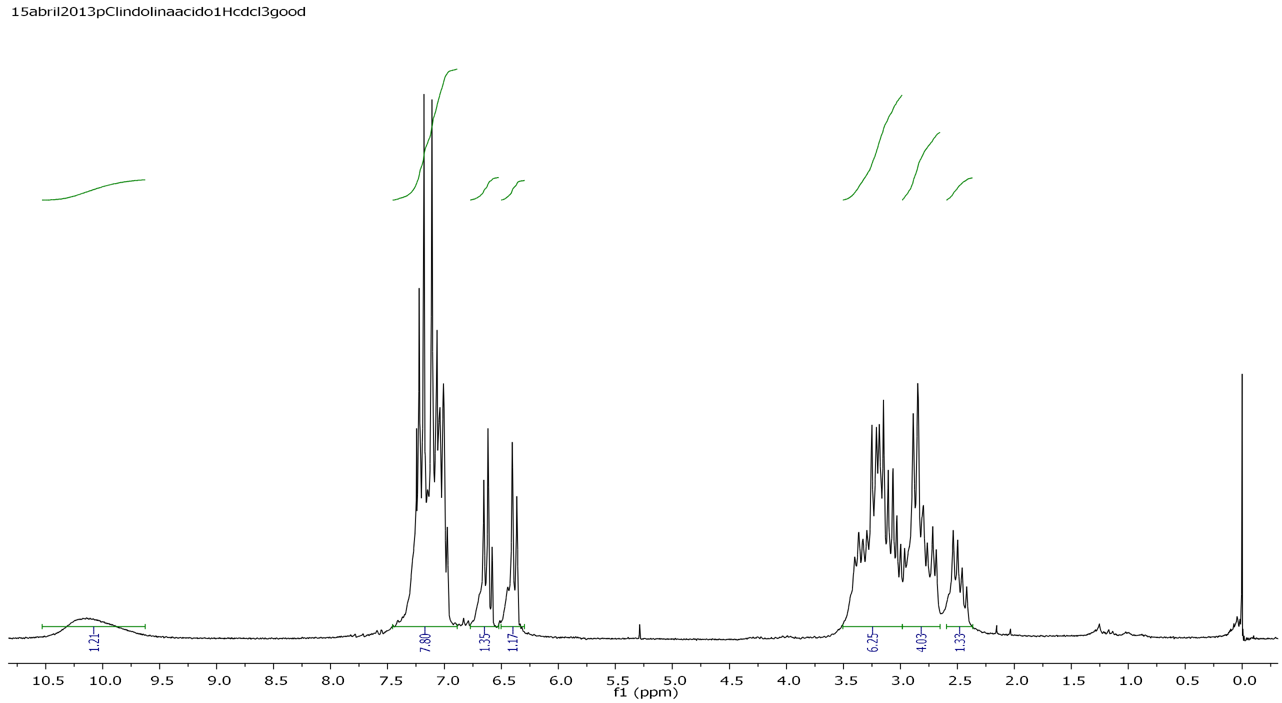

Figure S13. ^{1}H NMR (CDCl$_3$, 200 MHz) of **3-(4-Chlorophenyl)-4-(indolin-1-yl)butanoic acid (21b)**.

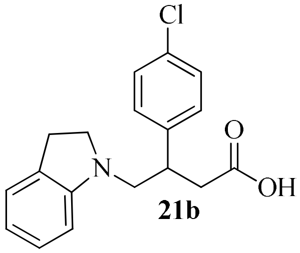

21b

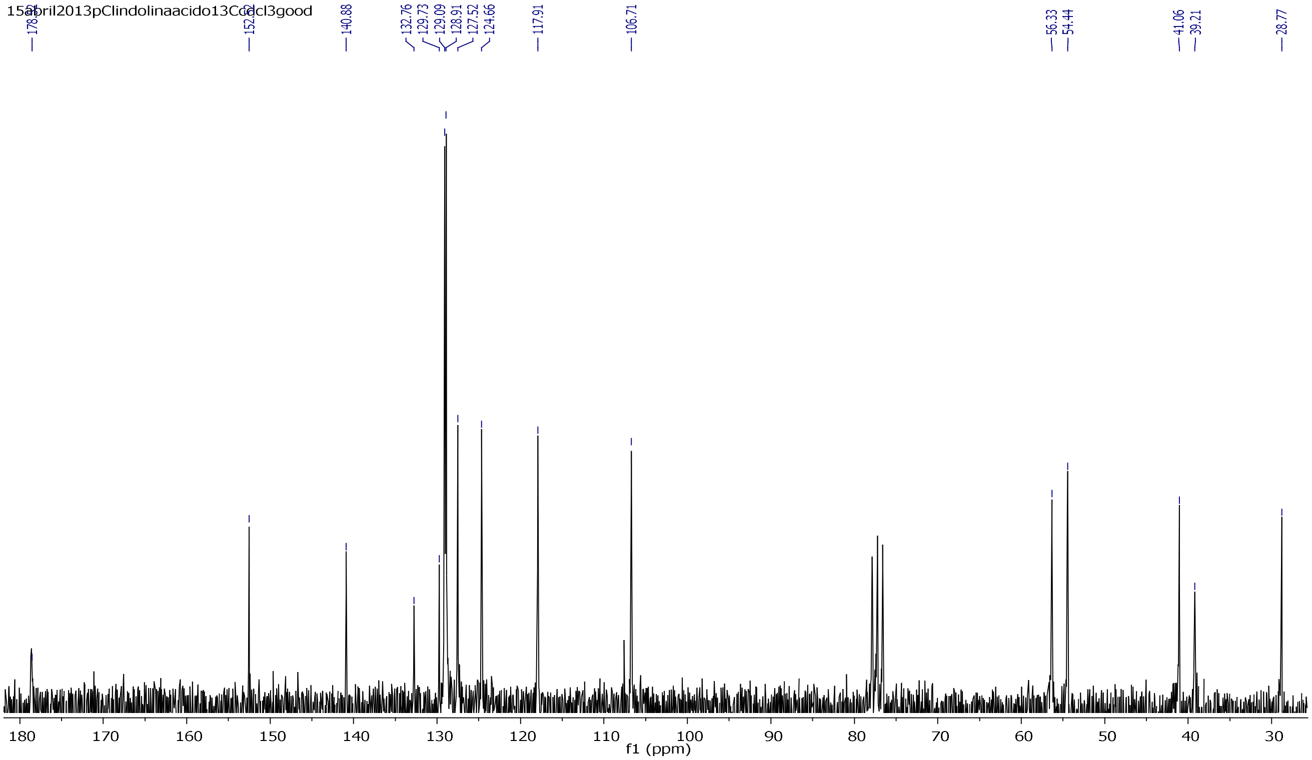

Figure S14. ^{13}C NMR (CDCl$_3$, 200 MHz) of **3-(4-Chlorophenyl)-4-(indolin-1-yl)butanoic acid (21b)**.

```
PF      ------------MSNKTNASLMKRREAAVPRGVGQIHP-IFAESAKNATVTDVEGREFID
EC      ---------------NSNKELMQRRSQAIPRGVGQIHPI-FADRAENCRVWDVEGREYLD
HS      -FDYDGPLMKTEVPGPRSQELMKQLNII--QNAEAVHFFCNYEESRGNYLVDVDGNRMLD
JB      -FDYDGPLMKTEVPGPRSRELMKQLNII--QNAEAVHFFCNYEESRGNYLVDVDGNRMLD

PF      FAGGIAVLNTGHLHPKIIAAVTEQLNKLTH---TCFQVLAYEPYVELCEKVNAK-VPGDF
EC      FAGGIAVLNTGHLHPKVVAAVEAQLKKLSH---TCFQVLAYEPYLELCEI-MNQKVPGDF
HS      LYSQISSVPIGYSHPALLKLIQQPQNASMFVNRPALGILPPENFVEKLRQSLLSVAPKGM
JB      LYSQISSIPIGYSHPALVKLVQQPQNVSTFINRPALGILPPENFVEKLRESLLSVAPKGM

PF      AKKTLLVTTGSEA--------------------------VENAVKIARATTGRAGVIAFT
EC      AKKTLLVTTGSEAVENA-------------------------VKIARAATKRSGTIAFS
HS      -SQLITMACGSCSNENALKTIFMWYRSKERGQRGFSQEELETCMINQAPGCPDYSILSFM
JB      -SQLITMACGSCSNENAFKTIFMWYRSKERGESAFSKEELETCMINQAPGCPDYSILSFM

PF      GAYHGRTMMTLGLTGKVVPYSAGMGLM--P-GGIFRALYPNELHGVS-V---DDSIAS-I
EC      GAYHGRTHYTLALTGKVNPYSAGMGL---MPGHVYRALYPCPLHGI----SEDDAIASI-
HS      GAFHGRTMGCLATTHSKAIHKIDIPSFDWPIAPFPRLKYPLEEFVKENQQEEARCLEEVE
JB      GAFHGRTMGCLATTHSKAIHKIDIPSFDWPIAPFPRLKYPLEEFVKENQQEEARCLEEVE

PF      ERIFKNDAEPRDIAAIIIEPVQGEGGFYVAPKAFMKRLRELCDKHGILLIADEVQTGAGR
EC      HRIFKNDAAPEDIAAIVIEPVQGEGGFYASSPAFMQRLRALCDEHGIMLIADEVQSGAGR
HS      DLIVKYRKKKKTVAGIIVEPIQSEGGDNHASDDFFRKLRDIARKHGCAFLVDEVQTGGGC
JB      DLIVKYRKKKKTVAGIIVEPIQSEGGDNHASDDFFRKLRDISRKHGCAFLVDEVQTGGGS

PF      TGTFFAMEQMGVAA--DLTTFAKSI-AGGFPLAGVCGKAEYMDAIAPGGLGGTYAGSPIA
EC      TGTLFAMEQMGVAP--DLTTFAKSI-AGGFPLAGVTGRAEVMDAVAPGGLGGTYAGNPIA
HS      TGKFWAHEHWGLDDPADVMTFSKKMMTGGFFH-----K-EEFRPNAPYRIFNTWLGDPSK
JB      TGKFWAHEHWGLDDPADVMTFSKKMMTGGFFH-----K-EEFRPNAPYRIFNTWLGDPSK

PF      CAAALAVMEVFEEEHLLDRCKAVGERLVTGLKAIQAKYPVI-GEVRALGAMIALELFEDG
EC      CVAALEVLKVFEQENLLQKANDLGQKLKDGLLAIAEKHPEI-GDVRGLGAMIAIELFEDG
HS      NLLLAEVINIIKREDLLNNAAHAGKALLTGLLDLQARYPQFISRVRGRGTFCSFDT----
JB      NLLLAEVINIIKREDLLSNAAHAGKVLLTGLLDLQARYPQFISRVRGRGTFCSFDT----

PF      DSHKPNAAAVASVVAKARDKGLILLSCGTYGNVLRVLVPLTSPDEQLDKGLAIIEECFSEL-
EC      DHNKPDAKLTAEIVARARDKGLILLSCGPYYNVLRILVPLTIEDAQIRQGLEIISQCFDEAK
HS      ----PDDSIRNKLILIARNKGVVLGGCGDKSIRFRPTLVFRDHHA--HLFLNIFSDILADFK
JB      ----PDESIRNKLISIARNKGVMLGGCGDKSIRFRPTLVFRDHHA--HLFLNIFSDILADFK
```

Figure S15 . Alignment of *pseudomonas fluorencens* (**PF**), *human* (HS), *E. coli* (EC) and *wild boar* (JB). Red and blue color letters corresponds to the residues of the chain A and chain B respectively, that interact with vigabatrin in the 1ohv crystal structure.

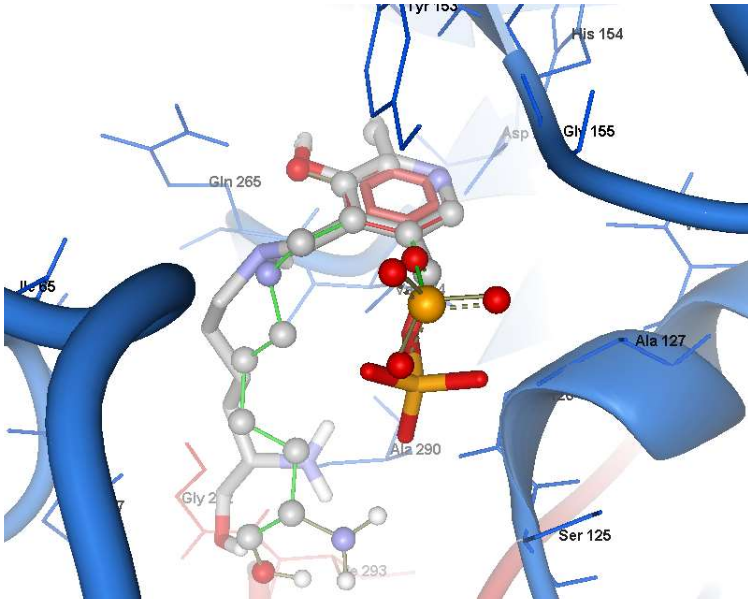

Figure S16. Validation of the molecular docking calculation for the pseudomonas model. Ligand in the 3r4t crystal structure was reproduced with a RMSD of 1.7 Å. Ligand experimental and calculated conformations are displayed as thick sticks and ball and stick representation respectively.

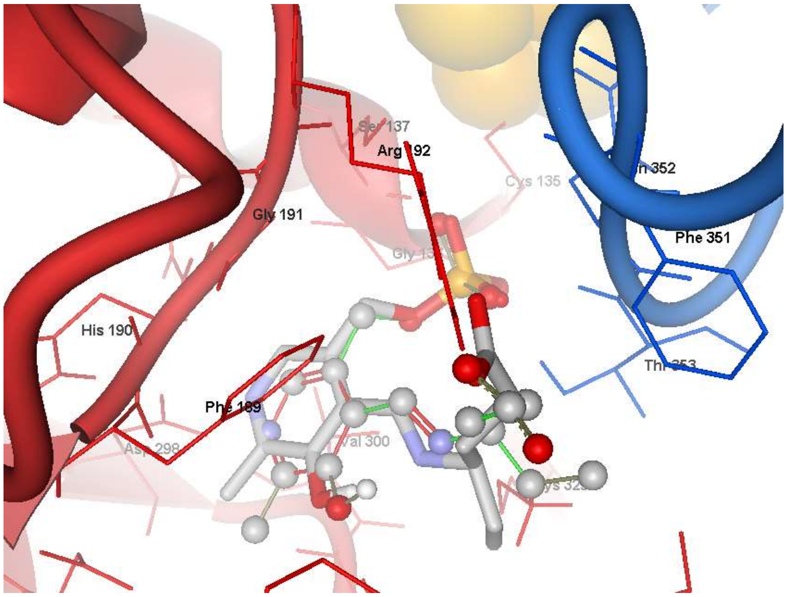

Figure S17. Validation of the molecular docking calculation for the *human* model. Ligand in the 1ohw crystal structure was reproduced with a RMSD of 1.3 Å. Ligand experimental and calculated conformations are displayed as thick sticks and ball and stick representation respectively.

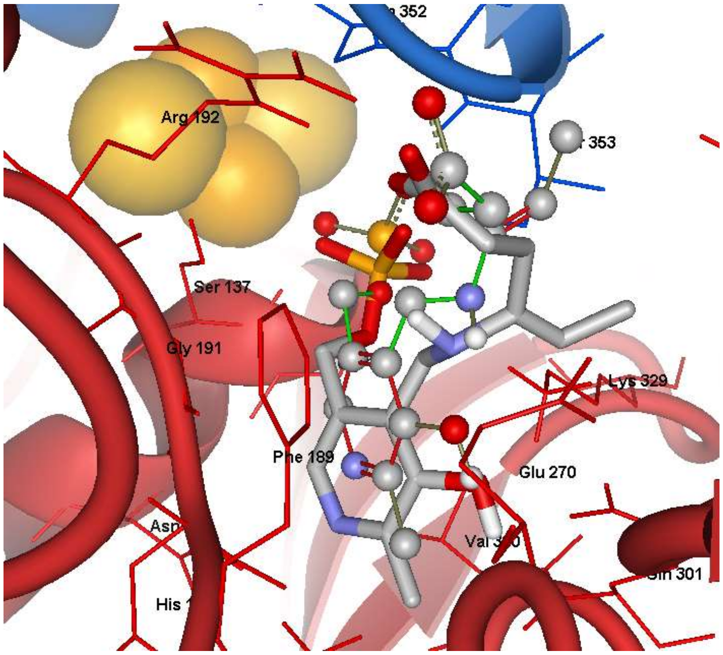

Figure S18. Validation of the molecular docking calculation for the *human* model. Ligand in the 1ohy crystal structure was reproduced with a RMSD of 1.8 Å. Ligand experimental and calculated conformations are displayed as thick sticks and ball and stick representation respectively.

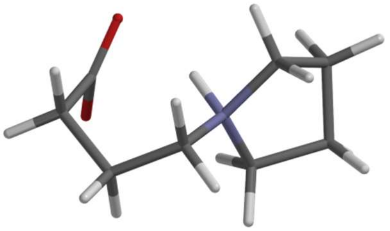

9a

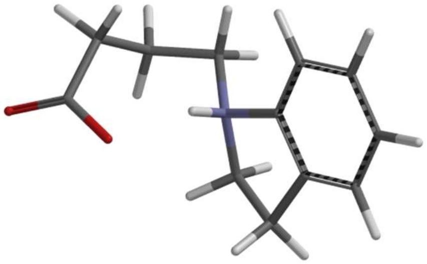

9b

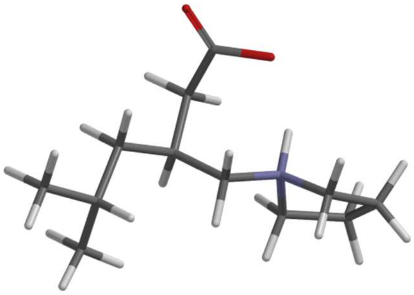

18a

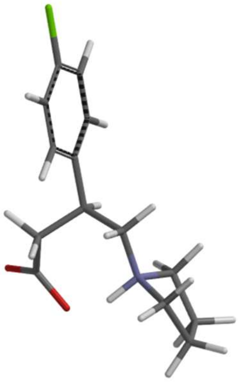

19a

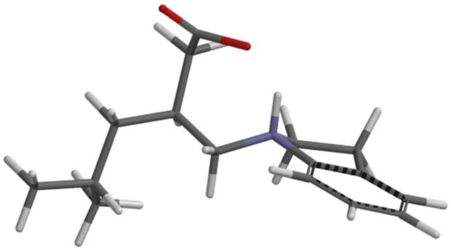

20b

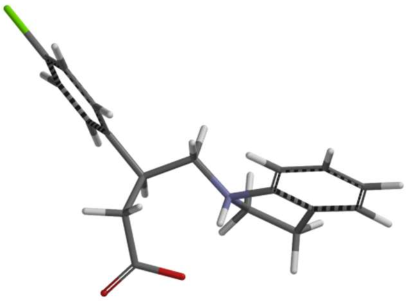

21b

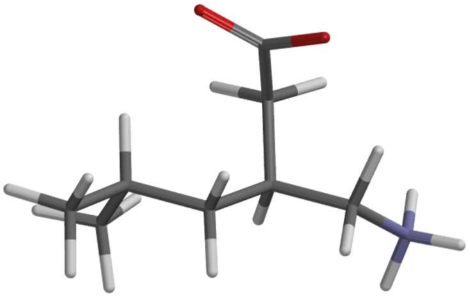

Pregabalin

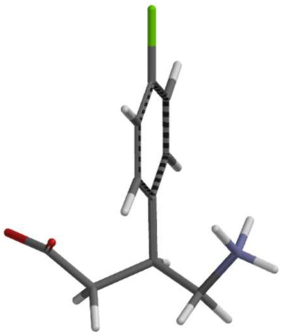

Baclofen

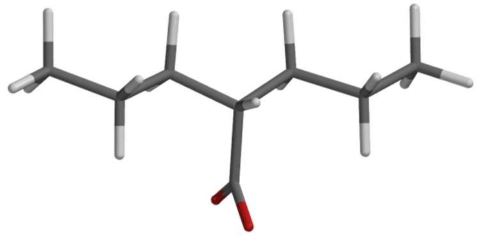

Valproate

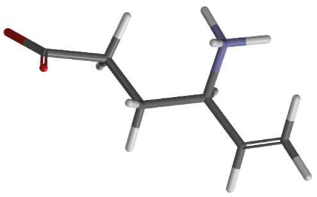

Vigabatrin

Figure S19. Optimized structures of all GABA analogues **9a**, **9b**, *(S)*-**18a**, *(S)*-**19a**, (*S*)-**20b**, *(S)*-**21b**, Baclofen, Pregabalin, Valproate and Vigabatrin molecules**.**

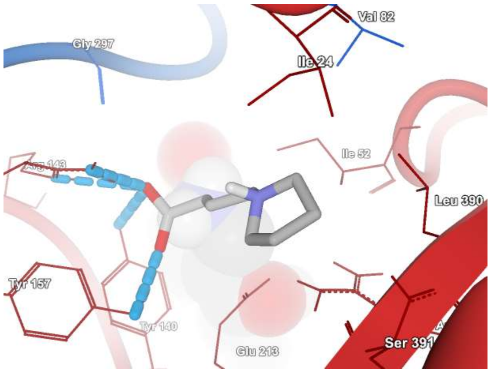

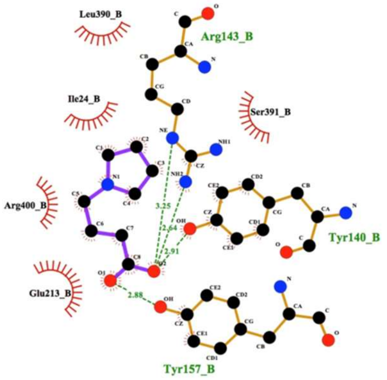

a)

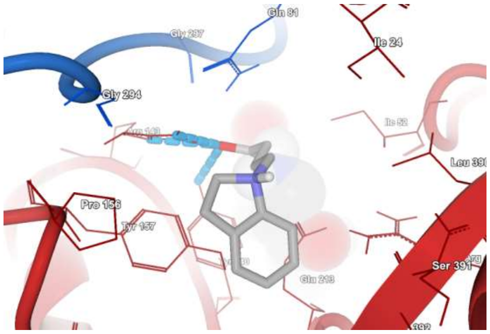

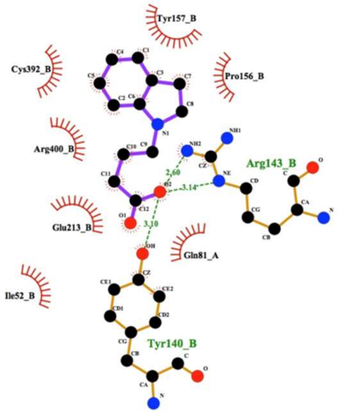

b)

Figure S20. a) **9a** hydrogen bond interactions (blue dashed lines) with pseudomonas GABA-AT in a 3D and 2D representation. b) **9b** hydrogen bond interactions (blue dashed lines) with pseudomonas GABA-AT. PLP prosthetic group is showed as spacefill model. The images were made with Molegro and LigPlot programs**.**

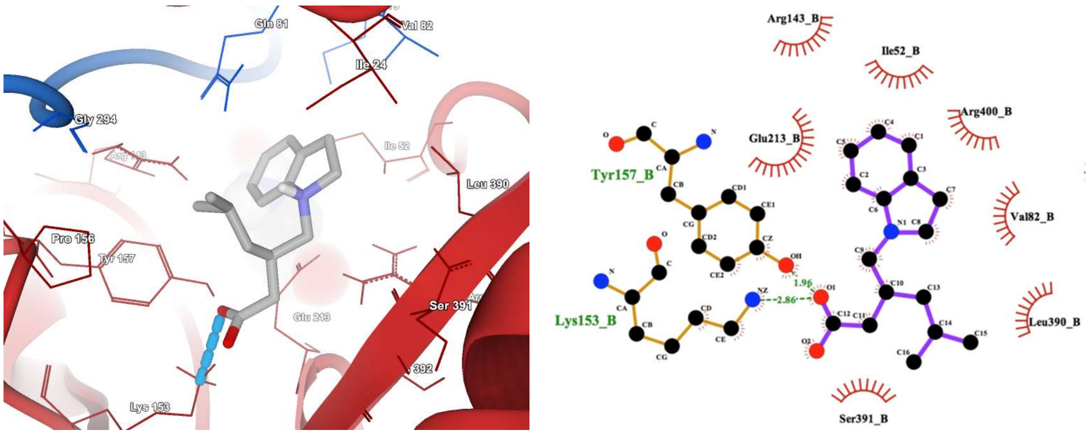

a)

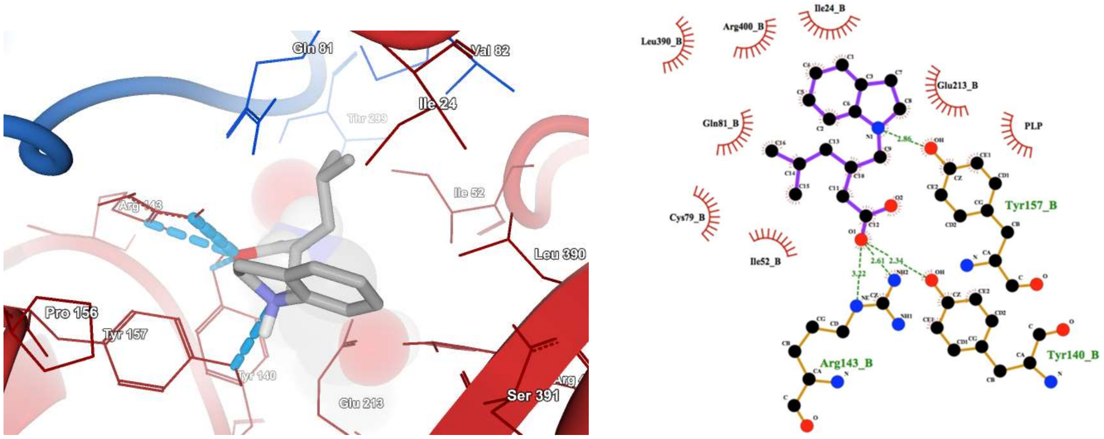

b)

Figure S21. a) (*R)*-**20b** hydrogen bond interactions (blue dashed lines) with pseudomonas GABA-AT in a 3D and 2D representation. b) *(S)*-**20b** hydrogen bond interactions (blue dashed lines) with pseudomonas GABA-AT. PLP prosthetic group is showed as spacefill model. The images were made with Molegro and LigPlot programs**.**

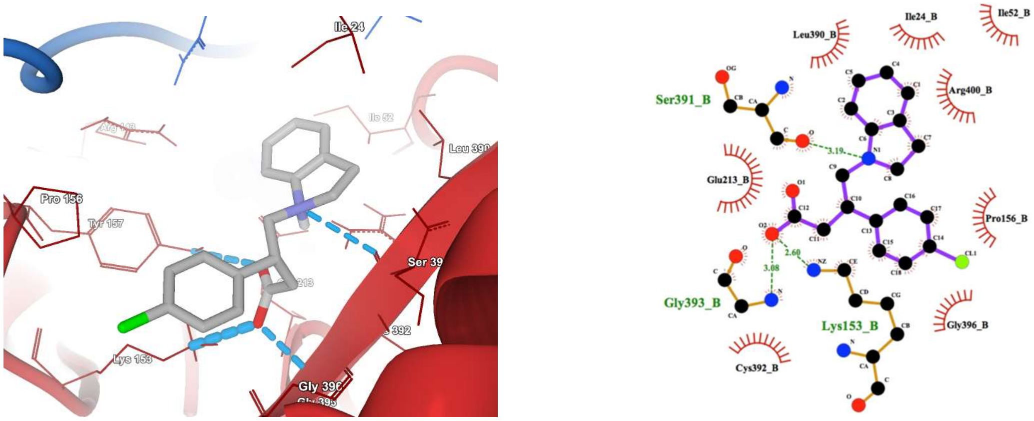

a)

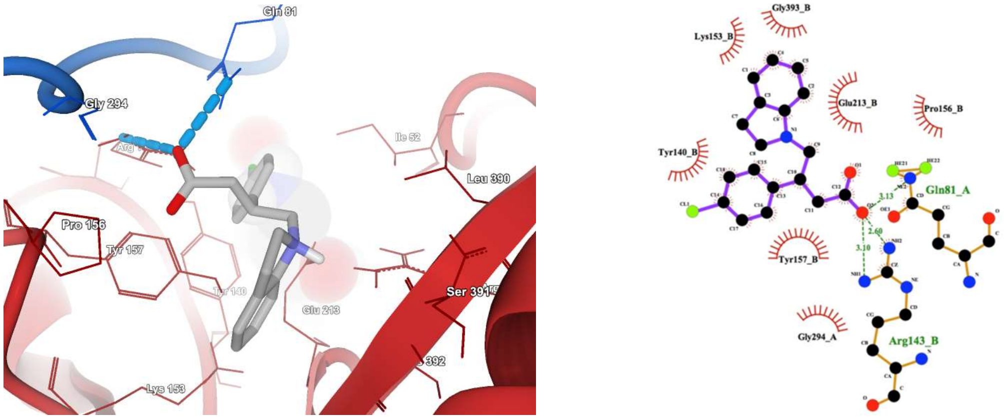

b)

Figure S22. a) (*R*)-**21b** hydrogen bond interactions (blue dashed lines) with *pseudomonas* GABA-AT in a 3D and 2D representation. b) (*S*)-**21b** hydrogen bond interactions (blue dashed lines) with *pseudomonas* GABA-AT. PLP prosthetic group is showed as spacefill model. The images were made with Molegro and LigPlot programs.

Table S1. Energy interactions values obtained from the docking calculations of all GABA derivatives and *pseudomonas* GABA-AT model. All the values are in kcal/mol.

Ligand	MolDock Score	Electro	HBond	Internal	LE
**9a**	-69.41	-2.39	-8.82	-2.17	-6.31
**9b**	-80.05	-1.13	-6.78	4.17	-5.34
(*R*)-**18a**	-71.30	-4.51	-0.08	-9.08	-4.75
(*S*)-**18a**	-82.63	-8.15	-6.96	-4.60	-5.51
(*R*)-**19a**	-27.88	-5.49	-1.97	-1.95	-1.55
(*S*)-**19a**	-91.07	-5.27	-6.65	-1.46	-5.06
(*R*)-**20b**	-81.00	-1.65	-5.55	1.87	-4.26
(*S*)-**20b**	-89.51	-6.91	-0.42	-2.89	-4.71
(*R*)-**21b**	-96.82	-4.44	-4.75	5.13	-4.40
(*S*)-**21b**	-102.18	-6.99	-6.50	1.64	-4.65

a) 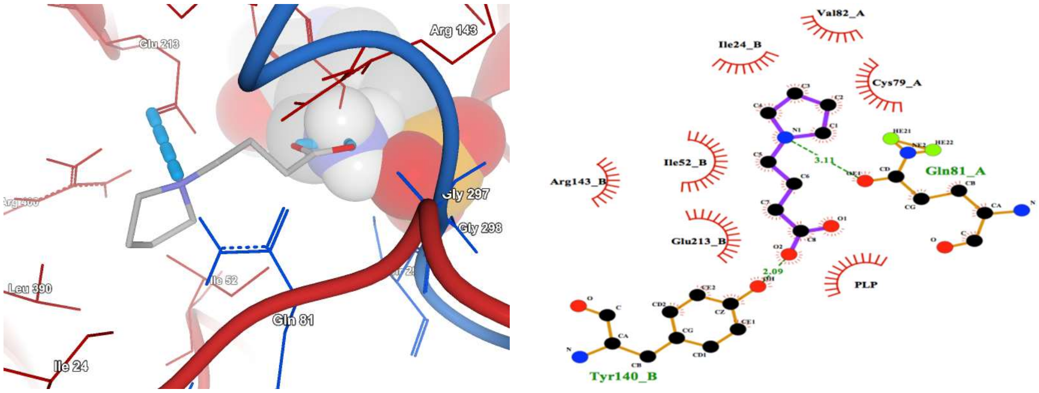

b) 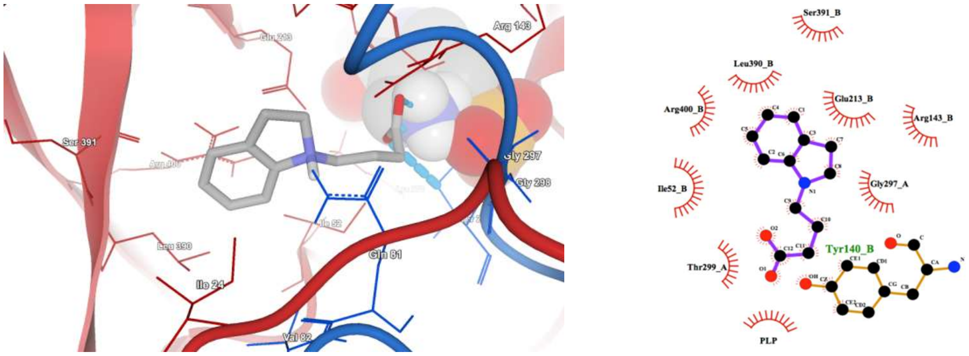

Figure S23. a) **9a** hydrogen bond interactions (blue dashed lines) with *human* GABA-AT in a 3D and 2D representation. b) **9b** hydrogen bond interactions (blue dashed lines) with *human* GABA-AT. PLP prosthetic group is showed as spacefill model. The images were made with Molegro and LigPlot programs.

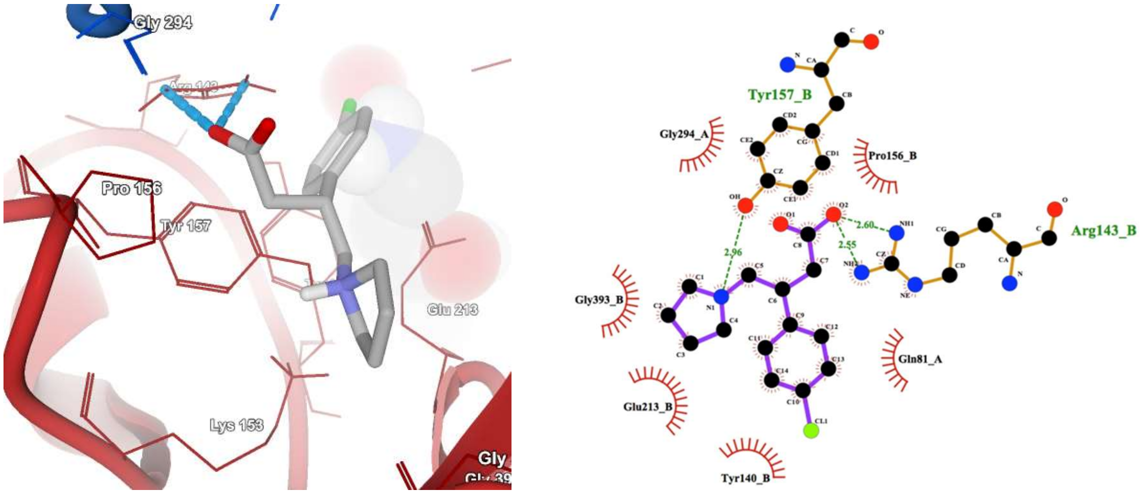
Gly 294
Pro 156
Tyr 157
Glu 213
Lys 153
Tyr157_B
Gly294_A
Pro156_B
Arg143_B
Gly393_B
Gln81_A
Glu213_B
Tyr140_B
2.96
2.60
2.55
a)

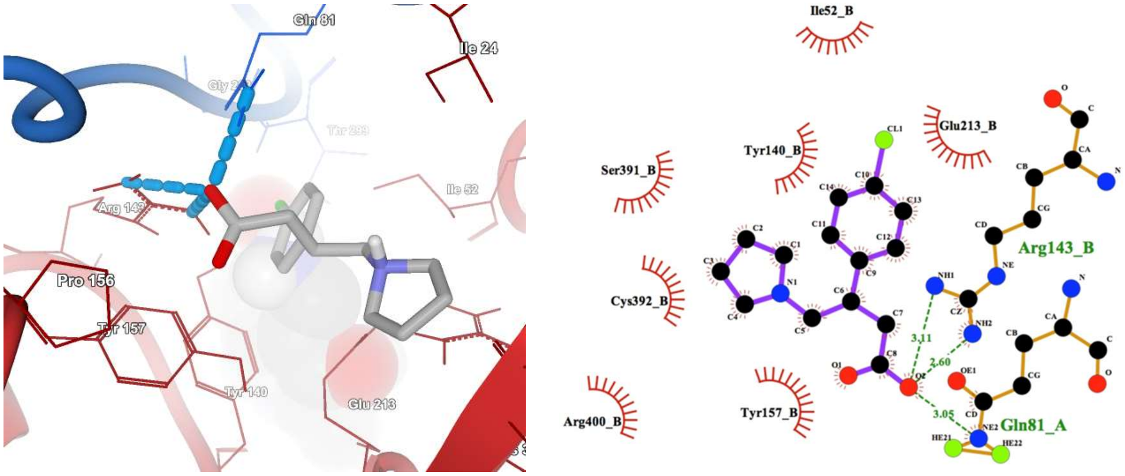
Gln 81
Ile 24
Ile 52
Pro 156
Tyr 157
Tyr 140
Glu 213
Ile52_B
Glu213_B
Tyr140_B
Ser391_B
Arg143_B
Cys392_B
Arg400_B
Tyr157_B
Gln81_A
3.11
2.60
3.05
b)

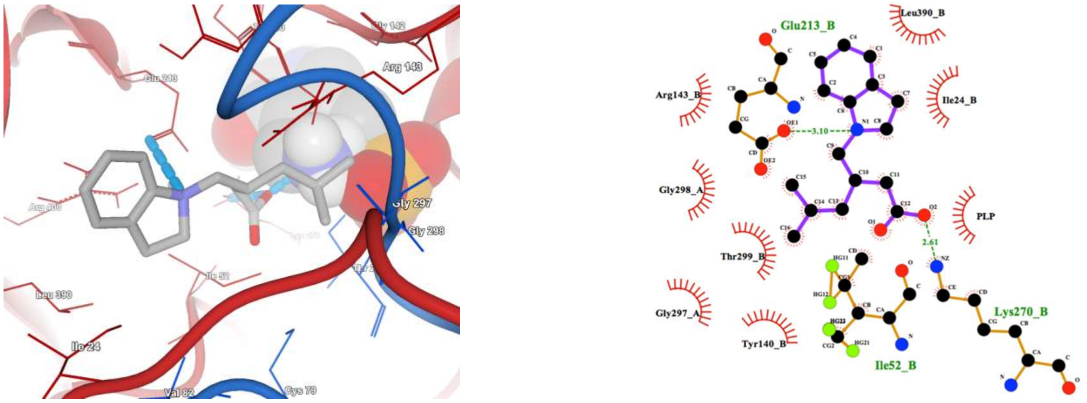
Arg 143
Gly 297
Gly 298
Leu 390
Ile 24
Val 82
Cys 79
Glu213_B
Leu390_B
Arg143_B
Ile24_B
Gly298_A
PLP
Thr299_B
Gly297_A
Tyr140_B
Lys270_B
Ile52_B
3.10
2.61
c)

d)
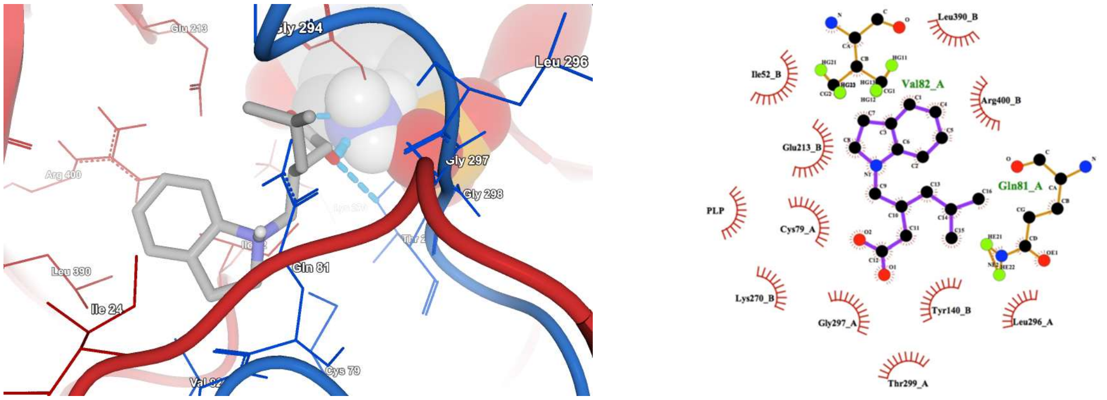

Figure S24. a) *(R)*-**18a** hydrogen bond interactions (blue dashed lines) with *human* GABA-AT. b) *(S)*-**18a** hydrogen bond interactions (blue dashed lines) with *human* GABA-AT. c) *(R)*-**20b** hydrogen bond interactions (blue dashed lines) with *human* GABA-AT in a 3D and 2D representation. d) *(S)*-**20b** hydrogen bond interactions (blue dashed lines) with *human* GABA-AT. PLP prosthetic group is showed as spacefill model. The images were made with Molegro and LigPlot programs.

a)
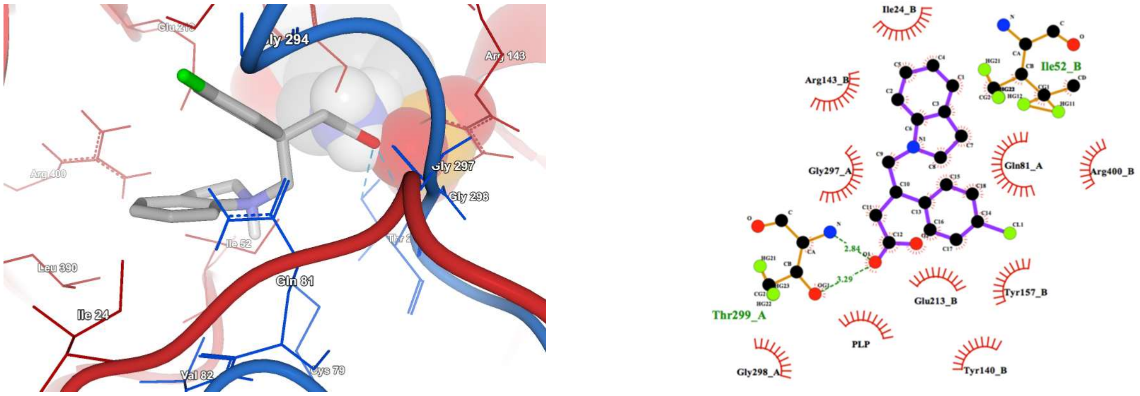

b)
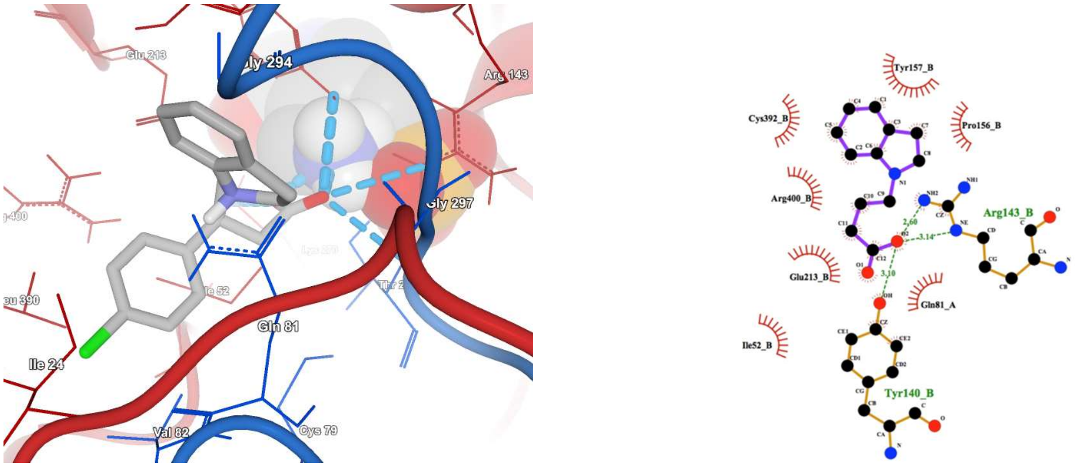

Figure S25. a) *(R)*-**21b** hydrogen bond interactions (blue dashed lines) with *human* GABA-AT in a 3D and 2D representation. b) *(S)*-**21b** hydrogen bond interactions (blue dashed lines) with *human* GABA-AT. PLP prosthetic group is showed as spacefill model. The images were made with Molegro and LigPlot programs.

Table S2. Energy interactions values obtained from the docking calculations of all GABA derivatives and *human* GABA-AT model. All the values are in kcal/mol.

Ligand	MolDock Score	Electro	HBond	Internal	LE
**9a**	-74.12	-2.31	-0.58	0.57	-6.74
**9b**	-91.25	-4.18	2.53	3.22	-6.08
(R)-**18a**	-76.06	-4.82	3.26	-3.15	-5.07
(S)-**18a**	-78.72	-5.35	-4.59	-6.57	-5.25
(R)-**19a**	-108.12	-5.32	4.23	3.721	-6.01
(S)-**19a**	-111.73	-1.97	-8.95	0.78	-6.21
(R)-**20b**	-80.93	-7.00	-4.94	1.72	-4.26
(S)-**20b**	-84.63	-0.61	-0.29	-2.00	-4.45
(R)-**21b**	-98.09	-0.94	0.42	-1.66	-4.46
(S)-**21b**	-21.54	-5.56	-5.72	6.96	-0.98

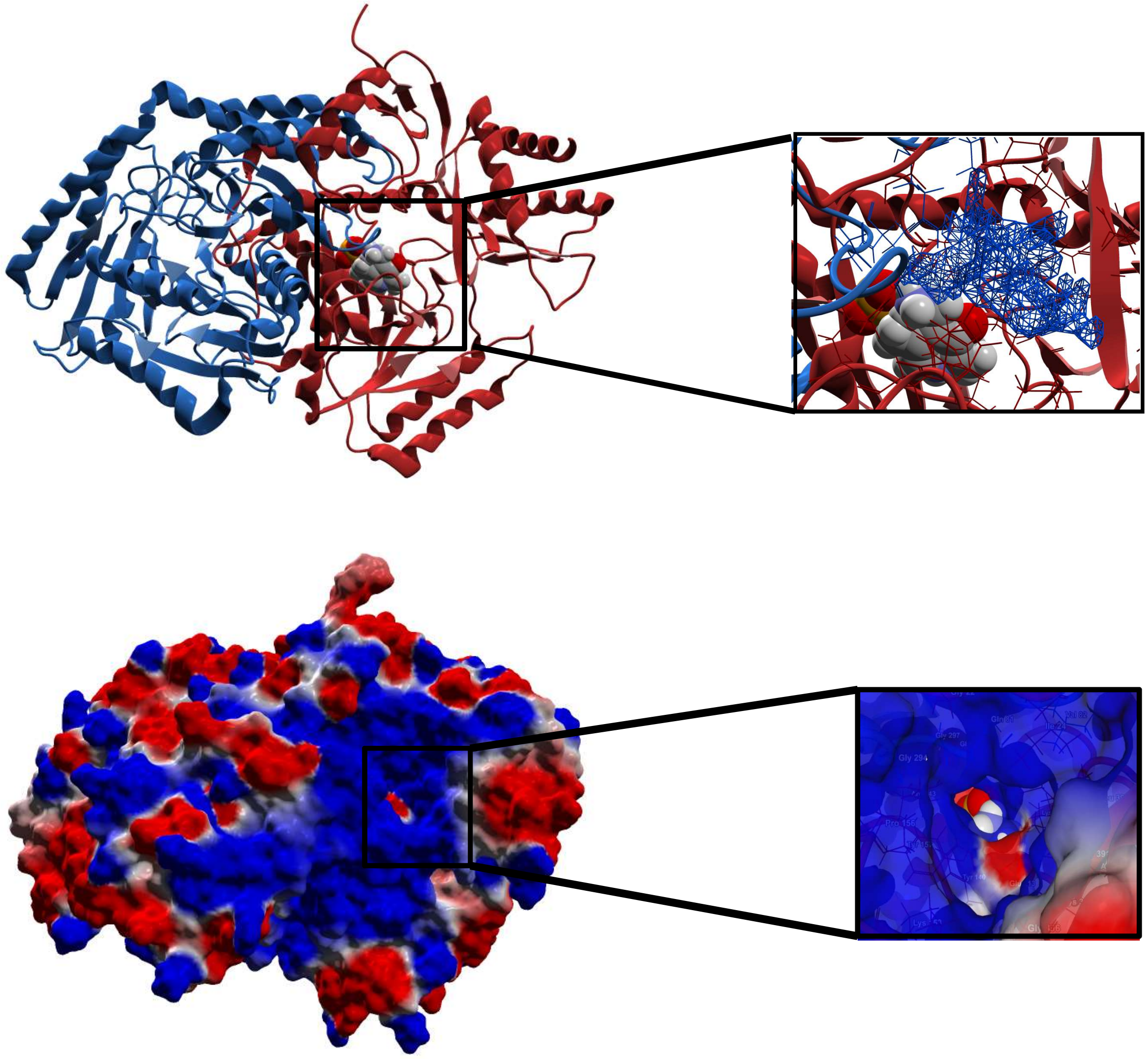

Figure S26. Homology model of *Pseudomonas* GABA-AT. The active site and protein cavity (blue color) is displayed. Electrostatic potential map of *Pseudomonas* GABA-AT. Blue, red and white colors represent regions with positive, negative and neutral electrostatic potential value, respectively. PLP prosthetic group is shown as spacefill model.

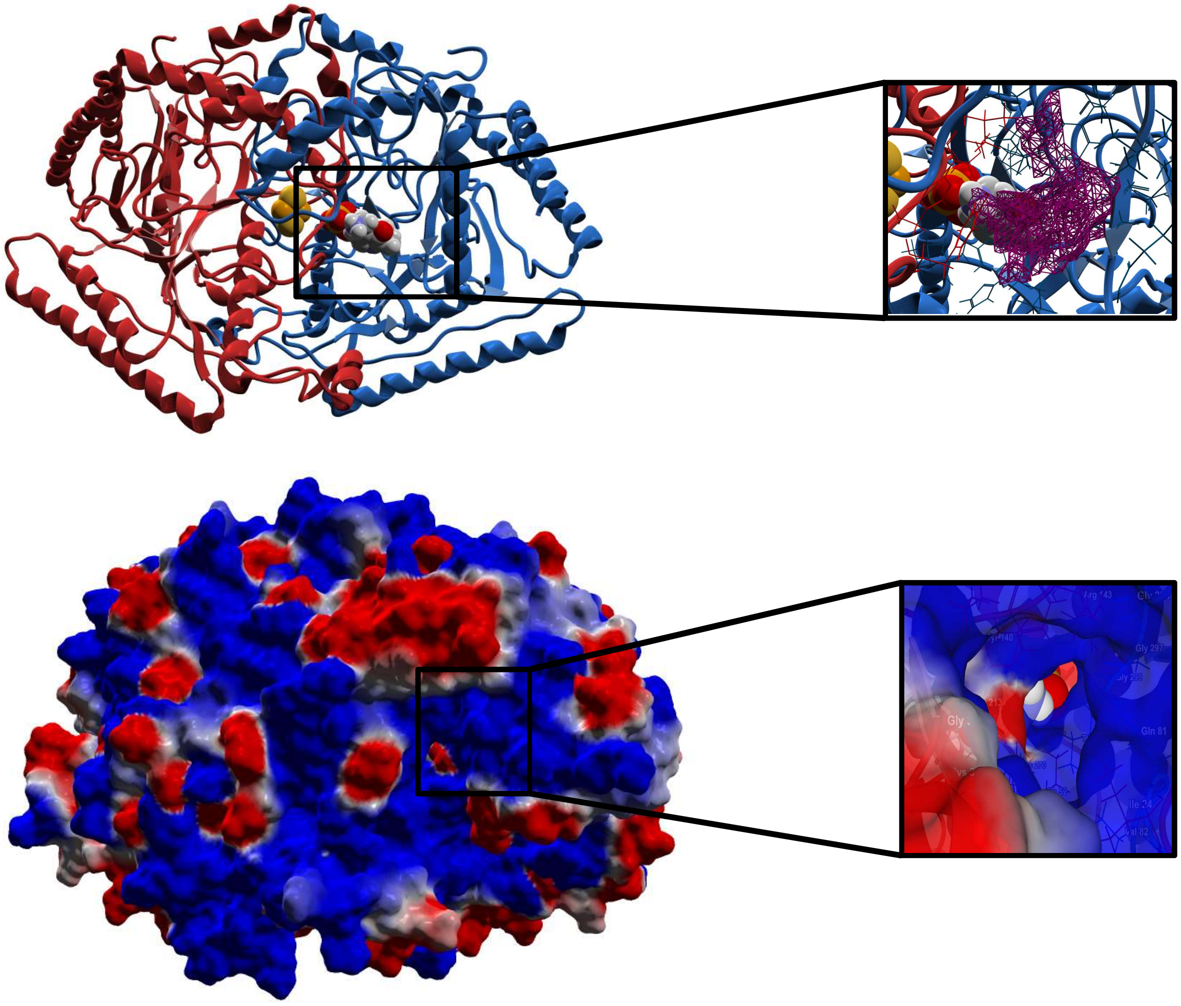

Figure S27. Homology model of human GABA-AT, the active site and protein cavity (purple color) is displayed. Electrostatic potential map of human GABA-AT. Blue, red and white colors represent regions with positive, negative and neutral electrostatic potential value, respectively. PLP prosthetic group and Fe_2/S_2 cluster are shown as spacefill model.
